# Mechanism of the Synergistic Toxicity of Ampicillin and Cefazoline on *Selenastrum capricornutum*

**DOI:** 10.3390/toxics12030217

**Published:** 2024-03-14

**Authors:** Feng-Ling Huang, Li-Tang Qin, Ling-Yun Mo, Hong-Hu Zeng, Yan-Peng Liang

**Affiliations:** 1 College of Environment Science and Engineering, Guilin University of Technology, Guilin 541004, China; huangfengling0901@163.com (F.-L.H.); zenghonghu@glut.edu.cn (H.-H.Z.); liangyanpeng@glut.edu.cn (Y.-P.L.); 2 Guangxi Key Laboratory of Theory and Technology for Environmental Pollution Control, Guilin University of Technology, Guilin 541006, China; molingyun123@126.com; 3 Collaborative Innovation Center for Water Pollution Control and Water Safety in Karst Area, Guilin University of Technology, Guilin 541006, China; 4 Technical Innovation Center of Mine Geological Environmental Restoration Engineering in Southern Karst Area, Nanning 530029, China

**Keywords:** β-lactam antibiotics, photosynthetic system, antioxidant system, transcriptomics analysis

## Abstract

Ampicillin (AMP) and cefazolin (CZO) are commonly used β-lactam antibiotics which are extensively globally produced. Additionally, AMP and CZO are known to have relatively high ecotoxicity. Notably, the mix of AMP and CZO creates a synergistic effect that is more harmful to the environment, and how exposure to AMP-CZO can induce synergism in algae remains virtually unknown. To yield comprehensive mechanistic insights into chemical toxicity, including dose–response relationships and variations in species sensitivity, the integration of multiple endpoints with de novo transcriptomics analyses were used in this study. We employed *Selenastrum capricornutum* to investigate its toxicological responses to AMP and CZO at various biological levels, with the aim of elucidating the underlying mechanisms. Our assessment of multiple endpoints revealed a significant growth inhibition in response to AMP at the relevant concentrations. This inhibition was associated with increased levels of reactive oxygen species (ROS) and perturbations in nitrogen metabolism, carbohydrate metabolism, and energy metabolism. Growth inhibition in the presence of CZO and the AMP-CZO combination was linked to reduced viability levels, elevated ROS production, decreased total soluble protein content, inhibited photosynthesis, and disruptions in the key signaling pathways related to starch and sucrose metabolism, ribosome function, amino acid biosynthesis, and the production of secondary metabolites. It was concluded from the physiological level that the synergistic effect of Chlorophyll a (Chla) and Superoxide dismutase (SOD) activity strengthened the growth inhibition of *S. capricornutum* in the AMP-CZO synergistic group. According to the results of transcriptomic analysis, the simultaneous down-regulation of *LHCA4*, *LHCA1*, *LHCA5,* and *sodA* destroyed the functions of the photosynthetic system and the antioxidant system, respectively. Such information is invaluable for environmental risk assessments. The results provided critical knowledge for a better understanding of the potential ecological impacts of these antibiotics on non-target organisms.

## 1. Introduction

Concerns regarding the potential contamination of water resources by various emerging organic contaminants are growing globally (EOCs) [1]. Pharmaceuticals are a fundamental group of EOCs, including antibiotics, antidiabetics, antiepileptics, antimalarial, analgesics, and anti-inflammatories, which are frequently reported in freshwater and marine environments [2,3,4]. Many of these drugs are not biodegraded during wastewater treatment and are discharged to aquatic environments in an active form [5,6,7]. Even at concentrations as low as µg L^−1^, pharmaceuticals have adverse effects, not only on the natural ecosystem, like fish and crustacea among others, but also on public health [8,9,10]. Feng’s study showed that β-lactam antibiotics, to a certain extent, delayed the time of the first laying of fleas and also the time of the first egg conception in *Daphnia magna* [8]. Pharmaceuticals have adverse effects on the growth and metabolism of microalgae, where they can disrupt photosynthetic and gene expression processes, increase the formation of reactive oxygen species, and change the activities of antioxidant enzymes [11,12,13]. The impact of these pharmaceuticals on the environment are more concerning, considering that they do not appear individually, but rather as a complex mixture, potentially leading to unwanted synergistic effects [14,15,16]. Beta-lactam antibiotics were one of the most widely used classes of antibiotics, largely due to their high therapeutic efficacy [17]. Ampicillin (AMP) and cefazolin (CZO) were the first widely prescribed synthetic beta-lactam antibiotics for the treatment of bacterial infections [18,19]. Due to its widespread use, AMP has been detected in quantities up to 27.1 μg L^−1^ (7.77 × 10^−8^ mol/L) in secondary wastewater [20].

Ampicillin (AMP) and cefazolin (CZO) have become the most frequently used antibiotics in hospital settings [21]. AMP and CZO are characterized by a high degree of persistence in the environment, leading to long-term ecotoxicity in various organisms [1]. The average ambient concentration of ampicillin and cefazolin detected in surface water samples was 0.7 ng/L–2.8 µg/L (AMP: 2.01 × 10^−12^ mol/L–8.02 × 10^−9^ mol/L; CZO: 1.54 × 10^−12^ mol/L–6.17 × 10^−9^ mol/L), which may relate to the location of the sample [22]. Ampicillin in water samples collected from Wastewater treatment plant samples were collected, and ampicillin was found in quantities ranging from 18 ng/L (5.1 × 10^−11^ mol/L) to 6.2 µg/L (1.78 × 10^−8^ mol/L) [23]. The concentration of cefazolin in the sewage treatment plant was larger, up to 12.85 µg/L (2.83 × 10^−8^ mol/L) [24,25]. The presence of AMP and CZO in the aquatic environment may have adverse effects on algae, fish, and crustacea, among others; these effects could include changing the structure of cell membranes and affecting photosynthesis [26,27]. Some antibiotics can interfere with the synergistic expression of the chloroplast genome, resulting in blocked chlorophyll synthesis; chlorophyll is affected, for example, by ampicillin and cefazolin [28]. Similarly, antibiotics can also induce the production of ROS in algae, thus causing severe oxidative stress and damage to cell membranes [29,30]. In order to explore the toxic effects of β-lactam antibiotics, which are widely used in the field of clinical medicine, some studies have studied the toxic effects of amoxicillin, ampicillin, penicillin, ceftazidime, and ceftriaxone on processes of photosynthesis in wheat leaves, and the results showed that antibiotics can inhibit photosynthesis [31].

Ampicillin and cefazoline are present in low concentrations in the environment, and they do have a negative impact on organisms in the aquatic environment. The 48 h-EC_50_ of ampicillin for *Microcystis aeruginosa* was 14.2 mg/L (4.07 × 10^−5^ mol/L) [32]. Many aquatic organisms live in environments polluted by a cocktail of toxic substances, including antibiotics. Despite this general assumption, the toxicity of different classes of antibiotics are examined separately in most cases [33]. Few studies have examined the toxicity of mixtures like the ones mentioned above [25]. The AMP acute toxicity is calculated as 160 mg·L^−1^, 172 mg·L^−1^, and 1530 mg·L^−1^ at 96 h for green algae, daphnid, and fish, respectively [2]. The EC_50_ of cefazolin to *S*. *capricornutum* is calculated as > 1000 mg·L^−1^ [34]. Although the toxicity of AMP and CZO when exposed to algae alone has been tested, no combined toxicity of the two has been reported [9]. In natural aquatic ecosystems, multiple classes of antibiotics are often detected concurrently [35,36,37]. Consequently, aquatic organisms may face exposure to antibiotic mixtures, where synergistic or antagonistic interactions may occur between antibiotics [38]. Despite this, the effects of such mixtures, particularly with regard to growth inhibition rates and biomass, have received limited attention in previous studies. *S. capricornutum*, a green alga, plays a pivotal role in oxygen production within aquatic ecosystems, occupying the lowest trophic level in food chains [39]. Due to its short culture cycle and its high sensitivity to related compounds, this alga can be used as a biotoxic model organism [40]. Changes in the diversity and abundance of *S. capricornutum* can have indirect yet significant repercussions on the broader aquatic community. The toxic effects of antibiotics on green algae are intrinsically linked to the inhibition and disruption of chloroplast metabolism. This, in turn, affects essential processes, such as photosynthesis and associated protein synthesis, ultimately impeding cell growth [14,41]. However, there is still a gap in the specific toxicity mechanism, particularly the study of the combined toxicity and synergistic mechanisms of AMP and CZO on algae.

This study aims to compare and evaluate the toxic effects of AMP and CZO on *S. capricornutum* at the physiological level, such as photosynthetic or antioxidant systems. Furthermore, to comprehensively understand and accurately compare the differing toxicity mechanisms of AMP and CZO on green algae, we employ RNA-seq analysis to investigate changes in differential expression genes (DEGs). The findings will contribute to our understanding of the toxicity mechanisms of β-lactam antibiotics in algae and their associated ecological risks.

## 2. Materials and Methods

### 2.1. Organism Test

The green alga *S. capricornutum* (algal species code: FACHB-271, discoverer: Printz, 1914) was purchased from the Freshwater Algal Library (FACHB) under the auspices of the Typical Culture Conservation Committee of the Chinese Academy of Sciences. To maintain the algal culture, a BG11 medium was utilized, and the culture was incubated in a constant light environment at 22 °C with an illuminance ranging from 2000 to 3000 Lux. The light–dark cycle was set at 12 h of light followed by 12 h of darkness [36,42]. Algal specimens in the logarithmic growth phase were selected for experimentation.

AMP (CAS No 69-53-4, molecular mass = 349 g/mol, slightly soluble in water) and CZO (CAS No. 25953-19-9, molecular mass = 454 g/mol, slightly soluble in water), both possessing a purity of 98%, were sourced from Shanghai Macklin Biochemical Technology Co., Ltd. (Shanghai, China). The preparation procedures of the AMP and CZO solutions were carried out using a sterile BG11 medium, with all procedures being conducted in sterile brown glass bottles.

### 2.2. Exposure Test Design

Under strict aseptic conditions, a homogeneous *S. capricornutum* algal culture, initially containing approximately 8 × 10^5^ cells·mL^−1^, was apportioned into 250 mL portions, with 20 mL of algae being dispensed into each Erlenmeyer flask. For each specific pollutant under investigation, a total of seven experimental groups and one negative control group were established, with each experimental group having three parallel groups. The AMP (3.47 × 10^−7^ mol/L–6.59 × 10^−5^ mol/L, dilution factor = 0.4) and CZO (1.37 × 10^−6^ mol/L-2.73 × 10^−4^ mol/L, dilution factor = 0.4) concentration ranges are based on the mixture test concentration and the mixing ratio. Following a 96 h exposure period, the growth inhibition ratio (*I*) was calculated for *S. capricornutum* in response to the two individual pollutants as well as their combination, utilizing the following formula [36,43,44,45,46]:

(1) $$I\;=1-\left({\mathrm{OD}}_{\mathrm{ti}}-{\mathrm{OD}}_{t0}\right)/\left({\mathrm{OD}}_{0i}-{\mathrm{OD}}_{00}\right)$$

where OD_t0_ and OD_ti_ are the optical density (OD) of the treatment at 0 and 96 h, respectively. OD_00_ and OD_0i_ are the OD of the controls at 0 and 96 h, respectively.

Based on the preliminary experiments, the binary mixtures (molar ratio: AMP:CZO = 0.20:0.80) were prepared using the equivalence fixed concentration ratio ray method. This approach is a multivariate mixture technique developed through the combination of the principles of the fixed concentration specific ray method and the fixed concentration effect method.

### 2.3. Toxicity Data Fitting

In the analysis of the concentration inhibition data, we applied the classical two-parameter nonlinear functions, specifically the Weibull function (Equation (2)) and the Hill function (Equation (3)). These mathematical models have been widely utilized in the field [6,36,44,47,48]. The selection of the optimal fitting function was based on either achieving the maximum determination coefficient (*R*^2^) or the minimum root-mean-square error (*RMSE*) [36].

(2) $$E\;=1-\exp\left(-\exp\left(\alpha +\beta \log_{10}\left(x\right)\right)\right)$$

(3) E=(α×x)/(β+x)

where *α* and *β* are the position and slope parameters in Equation (2), respectively; α and β represent the maximum effect and EC_50_ in Equation (3), respectively; *E* is the effect; and *x* is the concentration of an individual compound or mixture.

### 2.4. Toxicity Interactions of the Mixture

The concentration addition (CA, Equation (4)) [49] and independent model (IA, Equation (5)) [49] were used as the reference models for the evaluation of the mixture toxicity as follows:

(4) $$\sum_{i=1}^{m}\frac{c_{i}}{{\mathrm{EC}}_{x,j}}=1$$

(5) $$E(c_{mix})=1-\prod_{i=1}^{m}\left(1-E(c_{i})\right)$$

where *c*_i_ is the concentration of the *i*_th_ component in the mixture; EC_x,j_ is the corresponding concentration of *x*% effect when the *i*_th_ component exists alone; *E*(*c*_mix_) is the total effect of the mixture; *c*_mix_ is the concentration of the mixture; *E*(*c*_i_) is the effect of *i*_th_ component; and *c*_i_ is the concentration of *i*_th_ component.

In assessing the deviation from the reference models, we quantified the interactions of toxicity between the components of the mixture using a mixture deviation ratio (MDR) based on the CA and IA models [50,51]. The MDR is defined as the ratio between the predicted concentration based on the concentration addition or independent action (EC_x,Pred_) and the observed effect concentration (EC_x,Obs_), as represented by Equation (6).

For each effective concentration within the mixture, we calculated the lower (EC_xObs,lower_) and upper (EC_xObs,upper_) 95% observed confidence intervals (OCI) of the effect concentration. It is worth noting that there is no specific range of MDR values that signifies synergistic or antagonistic effects. The determination of the type of action is completed through the comparison of the MDR value and its upper and lower limits.

The calculations for the lower and upper 95% OCI of the MDR were performed according to Equations (7) and (8) as follows:

(6) $$\mathrm{MDR}=\frac{{\mathrm{EC}}_{x,\mathrm{CA}/\mathrm{IA}}}{{\mathrm{EC}}_{x,\mathrm{Obs}}}$$

(7) $${\mathrm{MDR}}_{\mathrm{upper}}=\frac{{\mathrm{EC}}_{x,\mathrm{Up}}}{{\mathrm{EC}}_{x,\mathrm{Obs}}}\;$$

(8) $${\mathrm{MDR}}_{\mathrm{lower}}=\frac{{\mathrm{EC}}_{x,\mathrm{Low}}}{{\mathrm{EC}}_{x,\mathrm{Obs}}}$$

where *x* is the effect (percentage) of a mixture, and MDR_lower_ and MDR_upper_ are the lower and upper 95%OCI of the MDR, respectively.

The following criteria were proposed to evaluate the toxicity interactions:

- MDR_lower_ < MDR < MDR_upper_ indicates an additive effect;
- MDR > MDR_upper_ corresponds to synergism;
- MDR < MDR_lower_ refers to antagonism.

### 2.5. Biochemical Biomarkers

Following a 96 h exposure in the algal toxicity experiments, 35 mL of algal culture was subjected to freeze centrifugation at 4 °C, with the centrifuge operating at 10,000 rpm for 10 min. After centrifugation, the supernatant was carefully discarded, and 1 mL of a phosphate-buffered saline (PBS) solution (0.05 mol·L^−1^, pH = 7.4, 4 °C) was added to the pellet. The mixture was pipetted into a 1.5 mL conical-bottom centrifuge tube, and subsequently subjected to rapid grinding at 75 Hz and 4 °C for 60 s using a fully automatic sample homogenizer. This homogenization process was repeated ten times.

The broken algal material was then centrifuged once more at 4 °C and 10,000 rpm for an additional 10 min. The resulting supernatant, known as the crude enzyme solution, was carefully collected and could be stored at low temperatures for subsequent enzyme activity analyses.

#### 2.5.1. Determination of Total Protein, Antioxidant Enzyme Activity, and Microreduced Glutathione

The crude enzyme solution served as the test sample and underwent analysis using several distinct methods. These methods included the Coomassie Brilliant Blue method as described by Bradford (1976), the ammonium molybdate method following the procedure outlined by Hugo Aebi (1984), the hydroxylamine method as detailed by Charles (1971), and a microplate test [33,52,53,54]. These assays were employed to quantitatively assess the total protein (TP) content, catalase (CAT) and superoxide dismutase (SOD) activities, and the glutathione (GSH) content, respectively.

The specific protocols and procedures utilized in these analyses were in accordance with established methodologies. The TP content was determined using a quantitative kit, the CAT activity was assessed with a visible light kit, the T-SOD activity was measured using a dedicated kit, and the GSH content was quantified with a dedicated kit. These kits were provided by the Nanjing Institute of Bioengineering (Nanjing, China).

#### 2.5.2. Determination of Malondialdehyde Content

A 5 mL aliquot of the algal solution underwent centrifugation at 8000 rpm for 10 min within a 4 °C centrifuge, followed by the careful removal of the supernatant. Each assay sample was subsequently treated with 2 mL of a 10% mass fraction trichloroacetic acid (TCA) solution and 2 mL of a 0.67% thiobarbituric acid (TBA) solution [55]. Following thorough mixing, each tube was immersed in a 100 °C water bath for 40 min. After cooling, another centrifugation step was performed at 8000 rpm for 10 min at 4 °C. To establish baseline measurements, three blank zeroing samples were prepared by combining 2 mL of the TCA solution with 2 mL of the TBA solution. The absorbance values of the supernatant at 450 nm, 532 nm, and 600 nm were determined using a UV-Vis spectrophotometer, and the Malondialdehyde (MDA) content was calculated based on the formulas outlined by Wu et al., 2020 [56].

(9) $$MDA=6.45({OD}_{532}-{OD}_{600})-0.56{OD}_{450}\times M/(1000\times N)$$

where OD_450_, OD_532_, and OD_600_ represent the absorbance at wavelengths of 450 nm, 532 nm, and 600 nm, respectively; M is the algae dilution factor; and N is the number of algal cells per milliliter.

#### 2.5.3. Determination of the Chlorophyll Content

The chlorophyll content was quantified using the ethanol extraction method, as detailed by Liu [41]. Following a 96 h exposure in the algal toxicity experiment, 20 mL of the algal solution was collected, frozen, and subsequently subjected to centrifugation at 4 °C and 10,000 rpm for 10 min, resulting in the removal of the supernatant. To extract the chlorophyll, 15 mL of a 95% ethanol solution was introduced. The samples were then sonicated for 20 min at 4 °C in a light-protected environment and left at 4 °C for an additional 24 h. After this incubation, the samples were shielded from light for a subsequent 24 h, followed by another round of centrifugation for 10 min.

Chlorophyll a (Chla), chlorophyll b (Chlb), and carotenoids (Car) were analyzed using a UV spectrophotometer at wavelengths of 470 nm, 649 nm, and 665 nm, respectively. The calculation formula is as follows:

(10) $$\mathrm{Chla}=13.59\;\times \;{\mathrm{OD}}_{665}-6.88\;\times \;{\mathrm{OD}}_{649}$$

(11) $$\mathrm{Chlb}=24.96\;\times \;{\mathrm{OD}}_{649}-7.32\;\times \;{\mathrm{OD}}_{665}$$

(12) $$\mathrm{Car}=(1000\;\times \;{\mathrm{OD}}_{470}-2.05\;\times \;{\mathrm{OD}}_{665}-114.8\;\times \;{\mathrm{OD}}_{649})/245$$

### 2.6. RNA-Seq Analysis

Following a 96 h exposure period, the maximum concentration point of the synergistic group (AMP-CZO), the concentration point for the corresponding individual substances (AMP, CZO), and the control group (CK) were chosen for RNA-seq analysis [57]. Each treatment was performed in triplicate for robust results. Subsequently, the respective samples were collected in 50 mL centrifuge tubes and subjected to centrifugation at 8000 rpm for 10 min. The supernatant was carefully removed, and the concentrated algal cells were promptly preserved by freezing them with liquid nitrogen. The samples were then transported under dry ice conditions to Guangzhou Kidio for testing and analysis. The specific testing and analysis process is described in Supplementary Figure S1.

### 2.7. Statistical Analysis

All tests were carried out in triplicate to provide averaged results. F tests are used in this paper and the data in this study show a normal distribution. One-way ANOVA was used to statistically analyze the differences between the three parallel experimental groups.

## 3. Results

### 3.1. Growth Inhibition of S. capricornutum Induced by AMP, CZO, and Their Mixture

**Figure 1:**
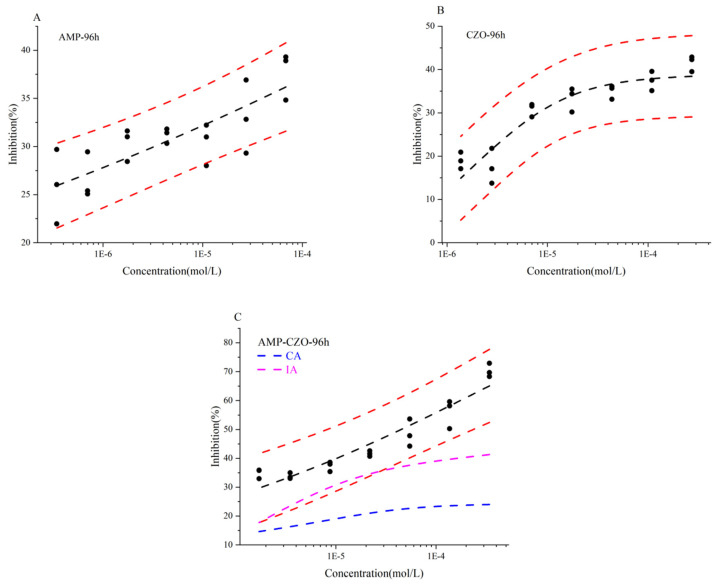
The toxicities of AMP, CZO, and AMP-CZO to *Selenastrum capricornutum* at 96 h. Note: Concentration addition (CA), Independent model (IA), 1E−5 = 1 × 10^−5^ and so on.

Different concentrations of each chemical exhibited varying inhibitory effects on the growth of *S*. *capricornutum* over a 96 h period. We employed one-way ANOVA to assess the variability among the three parallel groups, and all results demonstrated excellent reproducibility, with *p*-values exceeding 0.05, corresponding to a significance level (*α*) of 0.05. Concentration–response curves (CRCs) for AMP, CZO, and their mixture (AMP:CZO = 0.20:0.80) were depicted in Figure 1. Clear sigmoidal relationships between growth inhibition and concentration were observed, indicating a dose-dependent toxicity for AMP, CZO, and the AMP-CZO mixture. The assessment of toxicities using pEC_50_ allowed us to conclude that AMP, CZO, and AMP-CZO toxicity levels to *S*. *capricornutum* at 96 h were ordered as follows: AMP-CZO > AMP > CZO, with negative logarithm 50% effect concentration (pEC_50_) values of 4.41, 3.93, and 3.70, respectively.

The toxicity of the binary mixture of AMP and CZO far exceeded the expected simple additive (CA and IA) effects. To quantify the extent of synergy, based on MDR index results, MDR_CA_ = 1.03 and MDR_IA_ = 0.21 > MDRupper = 0.13. This strongly indicates a synergistic interaction between AMP and CZO, suggesting that their combined action triggers a greater growth inhibition effect than each antibiotic individually. The dose reduction index (DRI) results suggested that the toxicity of the AMP-CZO mixture on *S*. *capricornutum* was mainly contributed by AMP, as the DRI of AMP (23.10) was larger than that of CZO (13.18). Thus, the results revealed a synergistic interaction, where the combined impact of the antibiotics led to a significantly greater inhibition of *S*. *capricornutum* growth.

### 3.2. Impact of AMP, CZO, and Binary Mixtures on Algal Photosynthetic Pigments

**Figure 2:**
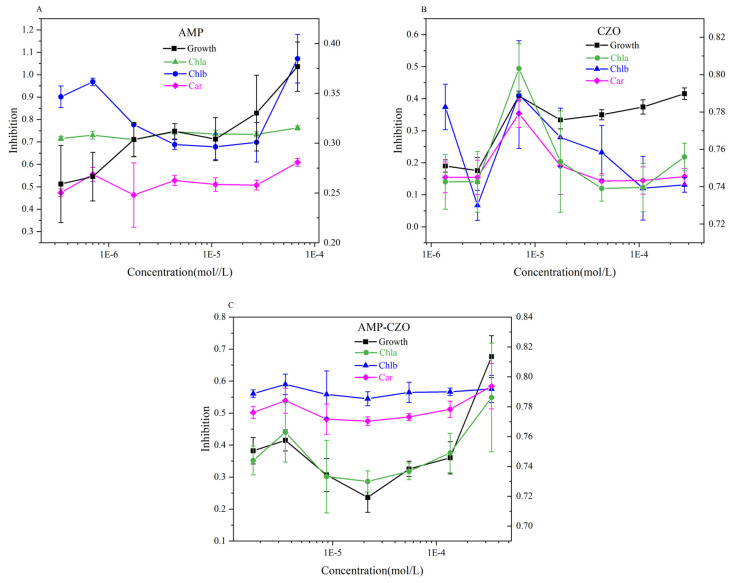
The changes in the photosynthetic pigment of AMP (**A**), CZO (**B**), and AMP-CZO (**C**) on *Selenastrum capricornutum.* Note: 1E−5 = 1 × 10^−5^ and so on. Growth = Growth inhibition, Chla = Chlorophyll a, Chlb = Chlorophyll b, Car = carotenoid.

Figure 2 showed the variations in the content of photosynthetic pigments, namely chlorophyll a, chlorophyll b, and carotenoids, which align with the results of the growth inhibition test. In the AMP experimental group, photosynthetic pigment contents showed a significant reduction across all tested concentrations (3.47 × 10^−7^ mol/L–6.82 × 10^−5^ mol/L) (*p* < 0.05). The highest growth inhibition (37.68%) occurred at 6.82 × 10^−5^ mol/L, corresponding to the lowest photosynthetic pigment content.

It can be clearly seen from Figure 2B that CZO has the most obvious inhibition of chlorophyll a in *S*. *capricornutum*, which is about 70%. The inhibition of *S*. *capricornutum* reached its peak at 6.98 × 10^−6^ mol/L; the inhibition rates of chlorophyll a, chlorophyll b, and carotenoids reached 80.32%, 41.31%, and 35.38%, respectively.

In the low-concentration region (1.71 × 10^−6^ mol/L to 2.18 × 10^−5^ mol/L), growth inhibition due to the AMP-CZO mixture declined with increasing treatment concentrations, while the photosynthetic pigment content increased. The 2.18 × 10^−5^ mol/L concentration marked an inflection point, wherein algal growth inhibition and photosynthetic pigment content both exhibited contrasting trends when compared to concentrations below 2.18 × 10^−5^ mol/L. Overall, however, chlorophyll a was most severely damaged at all test sites.

**Figure 3:**
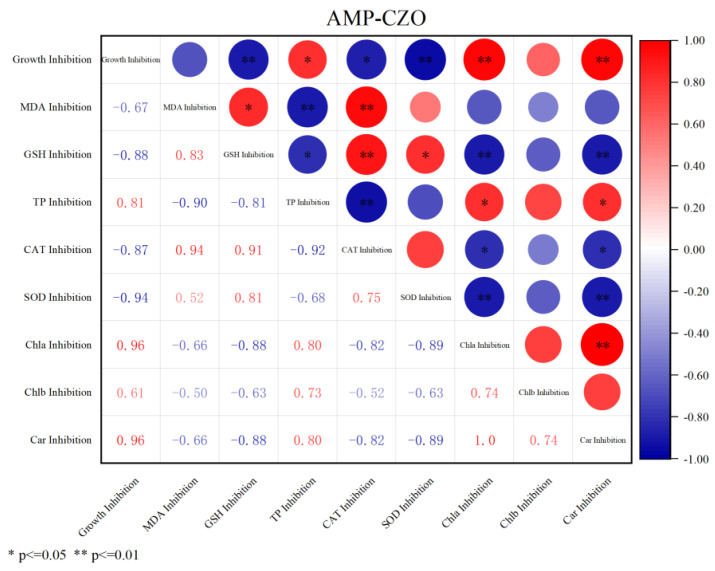
Pearson correlation analysis heat map of the biochemical indices of *Selenastrum capricornutum* to AMP-CZO following 96 h of exposure.

Regarding the changes in photosynthetic pigments, AMP-CZO had the most inhibited chlorophyll content of the three treatment groups (Table 1). According to the results of the MDR index, the chlorophyll a inhibition rate of the AMP-CZO treatment group produced a synergistic effect (MDR_CA_ = 1.82 > MDR_UPPER_ = 0.03, MDR_IA_ = 0.06 > MDR_UPPER_ = 0.03). This can also be seen from the inhibition rate of chlorophyll a following the three treatments. Figure 3 shows the correlation analysis of the physiological indicators of algae in the AMP-CZO treatment group. It was concluded that the growth inhibition rate of algae was significantly correlated with chlorophyll a and carotenoids, and the Pearson correlation coefficient was 0.96 (*p* < 0.01).

### 3.3. Impact of AMP, CZO, and Binary Mixtures on Algal Antioxidation Systems

**Figure 4:**
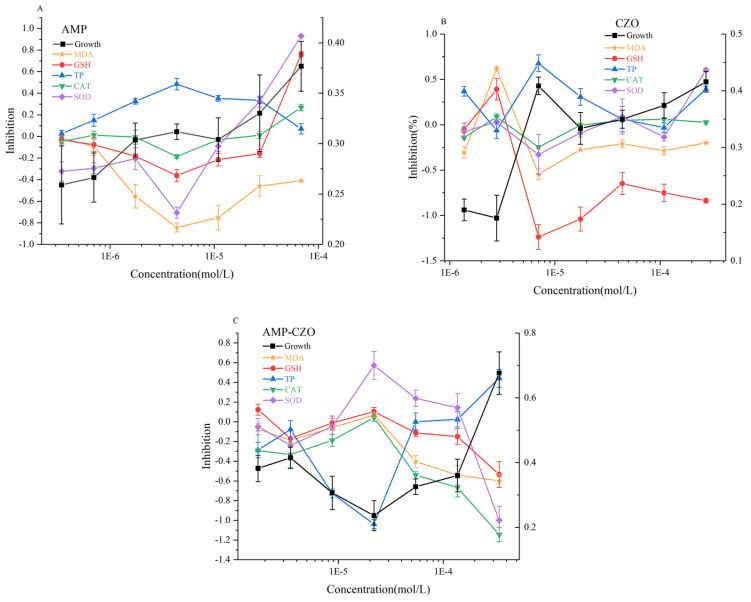
The inhibition of various indexes of AMP (**A**), CZO (**B**), and AMP-CZO (**C**) on *Selenastrum capricornutum.* Note: 1E−5 = 1 × 10^−5^ and so on. Growth = Growth inhibition, MDA = malondialdehyde, GSH = glutathione, TP = total protein, CAT = catalase, SOD = superoxide dismutase.

**Figure 5:**
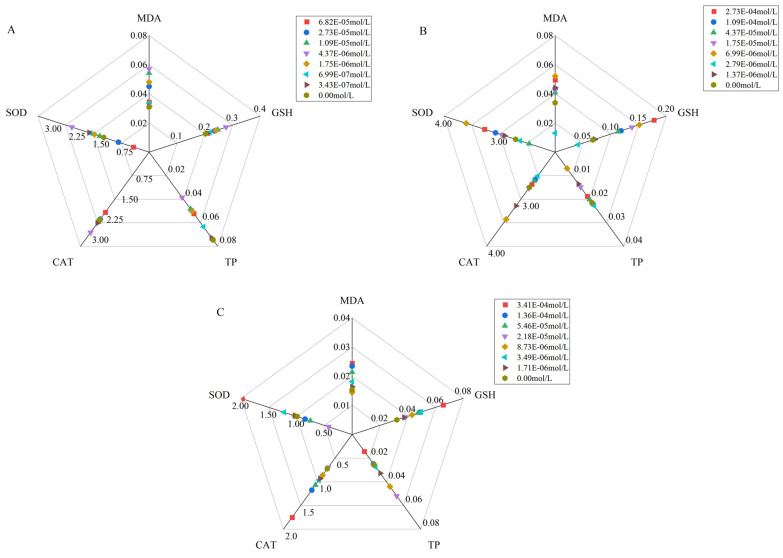
The change of algal enzymatic activities under AMP, CZO, and AMP-CZO exposure. (**A**) AMP; (**B**) CZO; (**C**) AMP-CZO. Each value was defined as the different content of each indicator. Note: 1E−5 = 1 × 10^−5^ and so on.

Under varying concentrations in each treatment group, the observed growth inhibition rates of algae corresponded with the measured indices, as illustrated in Figure 4. Figure 5A illustrated the effects of AMP on algal cells, revealing a significant decrease in the soluble protein content and an increase in the GSH content, SOD, CAT activities, and MDA content in the 3.47 × 10^−7^ mol/L to 6.82 × 10^−5^ mol/L AMP concentration range for 96 h (*p* < 0.05). MDA and GSH are basically in a state of stimulation, which together indicates that the algal cells are have been disturbed by an external force. SOD activity and CAT activity were inhibited at high concentrations (>2.73 × 10^−5^ mol/L), compared with the blank control group.

In the CZO treatment group, consistent with the occurrence of photosynthetic pigments, the stimulation of the MDA content, GSH content, CAT activity, and SOD activity showed high points at 6.98 × 10^−6^ mol/L, while the TP content was also the most inhibited. Not including 2.79 × 10^−6^ mol/L, the MDA content, GSH content, and SOD activity of *S*. *capricornutum* were inhibited at other concentration points. Notably, the point of the highest growth inhibition coincided with the minimum total protein content.

In the low-concentration range (1.71 × 10^−6^ mol/L to 2.18 × 10^−5^ mol/L), growth inhibition due to the AMP-CZO mixture exhibited a decreasing trend with an increasing treatment concentration, while the photosynthetic pigment content increased. The concentration of 2.18 × 10^−5^ mol/L marked a turning point, where algal growth inhibition, MDA content, GSH content, CAT activity, and SOD activity demonstrated an opposing trend to that observed at concentrations below 2.18 × 10^−5^ mol/L.

Table 1 shows that the growth inhibition rate of the AMP-CZO mixture on algae is significantly higher than that of the other two single substances. When compared with AMP and CZO, the synergistic mixture (AMP-CZO) had a significant inhibitory effect on the GSH of algae, and the SOD-CAT activity was −35.42% and −4.76%, respectively. According to the MDR index of the inhibition rate of SOD in algae following AMP-CZO exposure, SOD also played a synergistic effect (MDR_CA_ = 0.55 > MDR_UPPER_ = 0.34, MDR_IA_ = 0.86 > MDR_UPPER_ = 0.34). The total protein content of AMP-CZO was the only stimulus increase in the three test groups.

### 3.4. General Transcriptome Characteristics of S. capricornutum

To identify genes related to AMP, CZO, and the AMP-CZO mixture stress response in *S*. *capricornutum*, we constructed twelve transcription libraries, each with triplicates. These libraries comprised the control group (CK), AMP group (AMP-1), CZO group (CZO-1), and AMP-CZO group (AMP-CZO-1). Quality control assessments confirmed that all samples exhibited Q30 scores exceeding 91%, ensuring the data’s reliability. We obtained 24,660 unigenes; the assembly statistics are summarized in Table 2.

Unigene sequences were annotated and classified using BLAST software 2.6.0+ in Nr (19918), Swissprot (11619), KOG (9503), and KEGG (16615) databases (Figure S1). Notably, a significant proportion (65.88%) of Nr-annotated unigenes demonstrated substantial homology with *S*. *capricornutum*.

GO-term analysis classified the assembled unigenes into twenty-eight biological processes, three cellular components, and twenty molecular functions (Figure S2), with metabolic and cellular processes being prominent biological categories. The cellular anatomical entity featured as the dominant cellular component, while binding and catalytic activity were prominent molecular functions. These are all related to the growth of algal cells.

### 3.5. Differential Expression Analysis of Genes in S. capricornutum

We conducted an in-depth analysis of global gene expression levels using Illumina HiSeq. Prior to analysis, raw reads were filtered to ensure data quality. Clean reads were obtained from 12 mRNA libraries (Table 3), demonstrating good data quality and alignment with the genome and gene reference sequence. Principal component analysis (PCA) revealed a clear clustering of replicates within the same treatment group, as well as differentiation between different treatments. Specifically, PC1 and PC2 explained 63.1% and 15.2% of the variation, respectively (Figure S3A). The correlation levels between samples are presented in Figure S3B.

**Figure 6:**
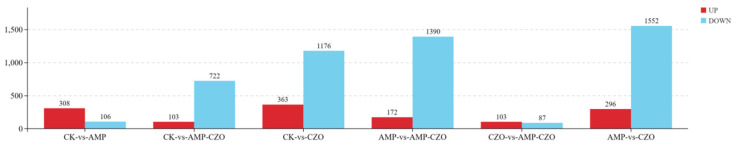
Number of differential genes.

DESeq2 analysis identified 414 significant differentially expressed genes (DEGs) when comparing AMP with CK, including 308 up-regulated and 106 down-regulated genes. In the comparison between CK and AMP-CZO, 825 DEGs were identified, consisting of 103 up-regulated and 722 down-regulated genes. Furthermore, a total of 1539 DEGs were detected in the CK vs. CZO comparison, comprising 363 up-regulated and 1176 down-regulated genes (Figure 6). These DEGs can indicate that the metabolism and growth of algae will be affected when exposed to AMP, CZO, and AMP-CZO mixtures.

### 3.6. Gene Enrichment Analysis

Significant GO terms, encompassing the molecular function (MF), the biological process (BP), and the cellular component (CC), are outlined in Figure S4A–C. In addition, our analysis revealed four, thirteen, and fourteen KEGG pathways that were significantly enriched (*p* < 0.05) in the AMP, CZO, and AMP-CZO exposure groups, respectively (Table 4).

The enriched KEGG pathways in the AMP treatment group included the nitrogen metabolism, photosynthesis-antenna proteins (Table 4 and Figure S5A), *nrt* (encodes nitrate/nitrite transporter), *nr* (encodes nitrate reductase), and *glnA* (encodes glutamine synthetase) of downward adjustments, which may disrupt the redox hemostasis, as exemplified by the excessive production of ROS within the algal cells. *Nrt*, *nr*, *glnA*, and synthesis genes for nitrite and amino acids are the main genes related to the nitrogen metabolism pathway. *Lhca1* and *lhca4*, which are photosynthesis-related genes for the synthesis of antenna proteins (encodes light-harvesting complex I Chl-a/b binding protein 1 and 4), are associated with the photosynthesis of *S. capricornutum*.

In the CZO exposure group, significant KEGG pathways comprised SNARE interactions in vesicular transport, ABC transporters, starch and sucrose metabolism, peroxisome, and carotenoid biosynthesis pathways (Table 4 and Figure S5B). *GPX1* (encoded glutathione peroxidase) and *sodA* are representative of the down-regulation of peroxisome, which affects the antioxidant system. *LCY1* (encoding lycopene beta cyclase), an important part of carotenoid synthesis, plays an important role in photosynthesis. The above genes are all involved in the biosynthesis pathway of algal cells.

Conversely, *S. capricornutum*, when exposed to AMP-CZO, exhibited enrichment in KEGG pathways, such as the biosynthesis of secondary metabolites and plant–pathogen interactions (Table 4 and Figure S5C). The down-regulation of *prp4* indicates the production of ROS, and the antioxidant system of the algae is affected as a result. The same goes for *glt1* and *E2.6.1.42*. *prp4*, *glt1*, and *E2.6.1.42* are related to the biosynthesis of secondary metabolites. *PSAK*, *PSAE* (encoding photosystem I reaction center subunit), and *LHCA4* (encoding chlorophyll A,B binding protein), all of which influence the metabolic pathways of algae, are indispensable parts of photosynthesis.

On the whole, the down-regulation of *LHCA4* (encoding chlorophyll A,B binding protein), *LHCA1,* and *LHCA5* (encoding light-harvesting protein of photosystem I) genes led to the blockage of chlorophyll synthesis in algae, which affected normal photosynthesis. The down-regulation of the *sodA* gene hinders the synthesis of peroxisomes and disrupts the antioxidant system. Together, the down-regulation of these genes leads to the creation of a synergistic effect that subsequentially inhibits *S. capricornutum* growth.

## 4. Discussion

### 4.1. The Toxicity of AMP and CZO on S. capricornutum

The toxicity of the antibiotics varies with the therapeutic class [47]. AMP (EC_50_ = 1.18 × 10^−4^ mol/L) belongs to the penicillin class of antibiotics, while CZO (EC_50_ = 1.99 × 10^−4^ mol/L) belongs to the cephalosporin class of antibiotics, and both have different toxicities after 96 h. Ampicillin plays a vital role in human and veterinary medicine for the treatment and prevention of diseases; its mechanism of action involves the disruption of peptidoglycan layer synthesis in the cell walls of a broad spectrum of bacteria [16]. Cefazolin is primarily indicated for the management of respiratory tract infections caused by Gram-positive bacteria [58]. The concentrations required for AMP, CZO, and AMP-CZO to achieve the same effect are different. For example, AMP (EC_50_ = 1.18 × 10^−4^ mol/L), CZO (EC_50_ = 1.99 × 10^−4^ mol/L), and AMP-CZO (EC_50_ = 3.89 × 10^−5^ mol/L). The EC_50_ of AMP and CZO obtained in this study are different from the existing studies, which may be due to the difference in the test organisms or the different exposure durations [1]. For example, the 48 h-EC_50_ of ampicillin for *Microcystis aeruginosa* was 14.2 mg/L (4.07 × 10^−5^ mol/L) [32].

### 4.2. The AMP-CZO Mixture Toxicity

Autotrophic microorganisms are continuously exposed to complex mixtures of substances, including antibiotics [55]. Therefore, it is vital to evaluate the potential interactions between the components of the mixture that could lead to a more significant outcome when compared to the impact of substances acting individually [58,59,60]. The risks associated with the presence of mixtures of contaminants may be significantly underestimated if we focus only on individual antibiotics. Previous research showed the apparent synergistic effect of binary mixtures of ciprofloxacin and other antibiotics on the growth of microalgae *Raphidocelis subcapitata* [61]. The same result was observed in the case of the combined toxicity of oxytetracycline, chlortetracycline, and enrofloxacin towards *Ankistrodesmus fusiformis* [62]. In the study of the toxicity of a mixture of sulfonamides, including sulfamethox azole and their transformation products to the microalgae Scenedesmus vacuolatus, simple additive effects were reported [63]. Another recent study demonstrated that a mixture of environmentally relevant concentrations of sulfamethoxazole and trimethoprim significantly reduced the growth of three marine microalgae species—*Nannochloropsis oculata*, *Chaetoceros neogracile*, and *Isochrysis galbana*—when compared to the effects of individual compounds [64]. Additionally, the nature of the combined toxicity can be influenced by both the exposure dosage and the mixture ratio [65]. The interactions between the antibiotics in the mixture varied from antagonistic to synergistic toxicity at a reversed ratio [66].

Notably, even low-level antibiotic pollution (in the range of ng/L to sub-μg/L) has the potential to act as a significant driver for promoting phytoplankton blooms, potentially leading to alterations in the structures and functions of aquatic food chains [67,68]. The presence of low, environmentally relevant concentrations (ng/L) of mixed antibiotics promoted the growth and photosynthesis rate, gene expression, and microcystin synthesis ability of cyanobacteria [20,69,70,71]. Similar effects were observed in the case of binary mixtures of low concentrations of spiramycin and ampicillin, resulting in intracellular microcystin synthesis and release stimulation [72]. A mixture of ciprofloxacin and sulfamethoxazole at a concentration below the toxicity threshold led to similar results, followed by strong proteomic responses and increased photosynthetic activity [73,74]. Microcystin production, photosynthesis, and growth stimulation could lead to the increased threat of cyanobacteria to the aquatic environment. The same is true for the effects of green algae. Moreover, as phytoplankton species are known to exhibit varying sensitivity to the xenobiotics, alterations of the biota composition and species succession are likely.

Our study, employing *S. capricornutum* as a model organism, investigated the impacts of AMP, CZO, and a combination of the two (AMP-CZO) on the transcriptional responses of this microalga. Our findings provide valuable insights into the underlying molecular mechanisms of toxicity and hormesis elicited by these substances, shedding light on critical pathways, such as lipid metabolism, energy metabolism, carbohydrate metabolism, and photosynthesis.

### 4.3. Molecular Mechanisms of AMP Toxicity

Transcriptomic responses have unveiled potential molecular mechanisms of AMP toxicity. To further investigate how *S. capricornutum* adapted to AMP environments at the molecular level, we analyzed the root transcriptomes of *S. capricornutum* when exposed to AMP. In this study, the most significantly enriched major pathways were nitrogen metabolism, carbohydrate metabolism, and energy metabolism.

#### 4.3.1. Regulation of Nitrogen Metabolism

Nitrogen is often a growth-limiting factor in plants and algae, and maintaining a balance between nitrogen metabolism and accumulation is crucial for preserving redox homeostasis [42,75,76]. We observed a consistent down-regulation of several genes, including *nrt* (encoding nitrate/nitrite transporter), *nr* (encoding nitrate reductase), and *glnA* (encoding glutamine synthetase). The down-regulation of these genes likely reduces the capacity of algae to utilize extracellular nitrate and nitrite as nitrogen sources in the production of intermediates for amino acid biosynthesis, as well as potentially disrupting redox homeostasis, as evidenced by the excessive production of ROS within algal cells. These results indicate that exposure to AMP modifies amino acid biosynthesis and metabolism, nitrogen metabolism, energy metabolism, and redox status, leading to a decrease in the total soluble protein content, ultimately inhibiting the growth of *S. capricornutum*. Specifically, both SOD and CAT activities were inhibited when compared to the control group. GSH levels were also affected, resulting in severe membrane damage, as confirmed by the stimulation of MDA levels.

#### 4.3.2. Response in Carbohydrate Metabolism

Carbohydrates serve not only as the primary energy source in organisms, but also, as signaling molecules, play a crucial regulatory role in plant growth, development, and stress responses [77,78]. Previous studies have indicated that the accumulation of soluble sugars, primarily sucrose, can enhance plant tolerance to abiotic stress and contribute to osmotic regulation in cells [79,80]. Additionally, the catabolism of glucose is believed to provide energy for plant growth, while reducing oxidative damage [81]. Furthermore, sugar alcohols can serve as intermediates in redox reactions or function as genuine ROS scavengers through hydrogen atom transfer mechanisms in order to counteract oxidative stress [80,82]. Our research demonstrated that the contents of sucrose, glucose metabolites (D-glucose 6-phosphate and D-fructose-6-phosphate), pentose phosphate pathway products (D-erythrose-4-phosphate), as well as cell wall-related metabolites (cellobiose) and sugar storage forms (D-maltose) significantly increased under AMP exposure. These results indicate that carbohydrate metabolism in *S. capricornutum* responds positively to AMP exposure via the providing of ATP and the removal of free radicals.

#### 4.3.3. Effect of Photosynthetic Pigment Level

Light capture is orchestrated by light-harvesting complexes, namely, LHC I and LHC II, which facilitate the transfer of absorbed excitation energy to the photosystem reaction centers PS I and PS II. LHC II plays a critical role in maintaining the balance of excitation energy between PS I and PS II, as well as in dissipating excess excitation energy [83]. Within these complexes, pigments are integrated into antenna proteins, forming the core components of light-harvesting complexes I and II (LHCI and LHCII), which are vital constituents embedded in the chloroplastic thylakoid membrane for effective light harvesting during photosynthesis [84].

In our study, we observed a down-regulation of genes encoding lhca1 and lhca4, which are responsible for light-harvesting complex I Chl-a/b binding protein 1 and 4. This down-regulation suggests that the exposure to AMP had a detrimental impact on the structure and function of LHCI. This impairment was associated with the excessive production of reactive oxygen species (ROS), such as HO• and ^1^O_2_. The LHC I and LHC II systems play a pivotal role in the efficiency of light capture. Chlorophyll a and carotenoids, which are the principal pigments in algae, are often used as indicators of algal growth and proliferation [85]. Consequently, the suppression of chlorophyll a, chlorophyll b, and carotenoid levels observed in our study may be attributed to the alterations in the LHC II system.

When the light energy absorbed by LHC II is transferred to the photosystem II (PS II) reaction center, the central pigment P680 initiates the generation of high-energy electrons, which are subsequently transferred to the original electron acceptor, thus leading to the photolysis of water [86]. These changes in the LHC II system significantly affected the growth and reproductive capacity of *S. capricornutum*.

### 4.4. Molecular Mechanisms of CZO Toxicity

#### 4.4.1. Transcriptomic Profiling and Pathway Alterations

Using a significance threshold of *p* < 0.05, we subjected 99 genes to KEGG pathway analysis, revealing significant enrichment in several pathways. Notably, the following aspects were affected: plant–pathogen interactions (fourteen genes down-regulated), starch and sucrose metabolism (twelve genes down-regulated), the peroxisome pathway (seven genes down-regulated), SNARE interactions in vesicular transport (six genes down-regulated), and the carotenoid biosynthesis pathway (four genes down-regulated).

#### 4.4.2. Oxidative Stress and Antioxidant Responses

In response to CZO exposure, we observed the initiation of defense mechanisms in *S. capricornutum*. The down-regulation of genes KSC1-KSC10, which are encoding defense proteins, led to the overproduction of reactive oxygen species (ROS). This initiated an oxidative stress response, characterized by a disrupted electron transport chain, which resulted in the accumulation of electrons that reacted with molecular oxygen, producing ROS as a result [87]. Specifically, the *sodA* gene exhibited a decrease in expression (Log_2_Fold change = −0.31), aligning with a significant reduction in superoxide dismutase (SOD) activity. Contrarily, the expression of the *CAT3* gene increased (Log_2_Fold change = 0.28), indicating a corresponding boost in catalase (CAT) activity. However, the presence of CZO significantly down-regulated the expression of the GPX1 gene (Log_2_Fold change = −1.21), leading to increased levels of reduced glutathione (GSH). Glutathione peroxidase (GPX), as the second line of defense against oxidative stress, catalyzes the conversion of excess hydrogen peroxide (H_2_O_2_) into water and the conversion of GSH into oxidized glutathione (GSSG), as well as reducing toxic peroxides to non-toxic hydroxyl compounds [88,89]. The concurrent down-regulation of *GPX1* and the up-regulation of *sodA* ultimately resulted in the accumulation of excessive H_2_O_2_, leading to oxidative damage in the cellular structure [90]. This effect was confirmed via elevated malondialdehyde (MDA) content and damage to the cell membrane. Previous studies have indicated that excessive ROS can impair the photosynthetic structure, disrupt energy transfer processes, and inhibit algal growth. Therefore, oxidative stress, induced by CZO exposure, serves as the secondary contributing mechanism to the growth inhibition of *S. capricornutum*. The reduced SOD activity leads to the persistence of superoxide radicals, thus further damaging the cell structure and causing a decline in GPX activity.

#### 4.4.3. Implications on Cellular Structures

Vesicular transport is responsible for the conveyance of proteins and lipids which are essential for various vital cellular activities in eukaryotic cells. This process encompasses the formation and maintenance of eukaryotic organelles, targeted protein transport, neurotransmitter release, and cell growth [91]. SNAREs constitute the core machinery that mediates membrane fusion, a crucial step in vesicular transport [92]. SNARE interactions in vesicular transport involve the movement of materials among Golgi bodies, endosomes, and lysosomes [93]. These organelles play diverse roles, including protein processing in Golgi bodies [94], the breakdown of proteins, nucleic acids, and polysaccharides in lysosomes [95], and the transport of extracellular materials into cells through endosomes [96]. The disruption of vesicular transport by volatile compounds may exert a significantly negative impact on organisms [46]. Therefore, the down-regulation of genes involved in protein metabolism and nutrient transport, as observed in our study, may account for the reduced total protein content and inhibition of *S. capricornutum* growth.

#### 4.4.4. Perturbation of Carotenoid Biosynthesis and Photosynthesis

Carotenoids, which are isoprenoids synthesized by organisms like plants, microalgae, animals, and cyanobacteria, are pivotal in harvesting light energy through the absorption of visible light during photosynthesis [97]. Carotenoids serve the crucial role of safeguarding photosynthetic bacteria, microalgae, and plants from light-induced cellular damage, in addition to their accessory functions [98,99]. In our study, the down-regulation of the LCY1 gene (lycopene β cyclase) likely contributes to the suppression of carotenoid levels in algae exposed to CZO. Lycopene, a carotenoid with robust singlet oxygen quenching and photoprotective activities, is believed to play a critical role in this regard [100,101]. The decrease in carotenoid levels negatively affects chlorophyll a and chlorophyll b, ultimately leading to an impairment of the photosynthetic electron transfer between QA and QB. This results in decreased photosynthetic efficiency and hampers the growth of algae.

### 4.5. Molecular Mechanisms of AMP-CZO Mixture Toxicity

#### 4.5.1. Metabolic Pathway Alterations and Amino Acid Biosynthesis

As illustrated in Figure S5C, the most impacted metabolic pathway is the “Biosynthesis of secondary metabolites”. Furthermore, according to the criteria of *p* < 0.05, the majority of differentially expressed genes were enriched in the “biosynthesis of amino acids” pathway.

Amino acids are fundamental components for all living organisms, including algae. They serve as building blocks for proteins, participating in various biological processes like growth, development, and reproduction [102,103]. Under stress conditions, there is a substantial increase in the content of amino acids and total proteins as a defensive mechanism [104,105]. This is evident in Figure 5C, which shows an improvement in the total protein content relative to the control group. This further illustrates that the defense mechanism of the algae has been forced to activate under the treatment of the AMP-CZO mixture. At this time, the growth of algae is significantly inhibited. Proline, a key amino acid, plays a crucial role in mitigating stress-induced damage via the regulation of the cellular osmotic balance, scavenging reactive oxygen species (ROS), and maintaining membrane integrity [106].

The down-regulation of *prp4*, encoding a proline-rich protein, results in an accumulation of ROS and a disruption of membrane integrity. This upsurge in ROS levels triggers an enhancement in the activity of superoxide dismutase (SOD) and catalase (CAT), which represent the first line of defense against oxidative stress. In parallel, the content of malondialdehyde (MDA), an indicator of lipid peroxidation, increases in response to AMP-CZO exposure. The AMP-CZO mixture reduced the proline content of algae, which in turn affected the biosynthesis of amino acids (secondary metabolites), consequently leading to the alteration of metabolic pathways.

#### 4.5.2. Impact on Amino Acid Biosynthesis and Carbon Metabolism

It is crucial to note that the metabolism of amino acids is closely linked to other metabolic pathways, such as central carbon metabolism, nitrogen metabolism, and sulfur metabolism [75]. The down-regulation of genes such as *got1* (cytoplasmic aspartate aminotransferase), *asp5* (chloroplastic aspartate aminotransferase), *glt1*, and *E2.6.1.42* possibly contributes to the reduced levels of L-glutamate, glutamine, L-leucine, and isoleucine. This down-regulation of amino acid biosynthesis suggests potential impairments in the carbon metabolism and energy production of algae, leading to their diminished growth performance. Carbon metabolism pathways include ascorbic acid and aldehyde metabolism, glycolysis/gluconeogenesis, fructose, and mannose metabolism. Furthermore, sulfur is essential for the synthesis of glutathione (GSH), which plays a vital role in antioxidant defense. Therefore, the reduced accumulation of GSH might be attributed to disturbances in sulfur metabolism [107], potentially due to the damage caused to the sulfur absorption in *S. capricornutum* under AMP-CZO exposure. The decrease in GSH content leads to the accumulation of ROS.

Excessive ROS production inhibits the synthesis of chlorophyll b, an auxiliary pigment that plays a crucial role in light absorption and energy transfer during photosynthesis [45]. Chlorophyll b captures light energy and converts it into electrical and chemical energy via a series of electron transfer reactions involving chlorophyll a [27,69,108]. The production of chlorophyll a and chlorophyll b is blocked, and the photosynthesis pathway of algae is blocked.

#### 4.5.3. Photosynthetic Impairment and Growth Inhibition

The impairment of chlorophyll a and b significantly interferes with the photosynthetic electron transport between QA and QB, ultimately damaging the efficiency of the photosystem II (PSII) system. As a result, various photosynthetic parameters, such as carotenoid levels, are reduced [109,110]. The down-regulation of the *LHCA4* gene also proves to be a phenomenon. In response to such damage, algal cells attempt to repair the photodamaged proteins (*PSAK* and *PSAE*) within the PSII by replacing them with newly synthesized subunits. However, if the rate of repair cannot keep up with the rate of photoinactivation, the PSII pool experiences a net photoinactivation, leading to a decrease in the photosynthetic quantum yield [111,112]. The inhibition of PSII activity in this study is likely due to the rate of photodamage exceeding the rate of repair. Consequently, the suppression of algal photosynthesis directly contributes to the inhibitory effect on algal growth.

In conclusion, the main source of damage to the *S. capricornutum* under exposure to the AMP-CZO synergistic group was the obstruction of the amino acid biosynthesis pathway. The resulting changes in metabolic pathways such as carbon metabolism and sulfur metabolism also affect the normal operation of the photosynthetic system.

## 5. Conclusions

The comprehensive assessment of multiple apical endpoints at the populational, physiological, and transcriptional levels, as conducted in the present study via the employment of a systems biology approach, can provide detailed mechanistic insights into chemical toxicity. In addition, the findings offer a new development in the toxicological milestones of water ecosystems.

Our study highlighted the dose-dependent toxicity of AMP, CZO, and their mixture (AMP:CZO = 0.20:0.80) on *S. capricornutum* growth, with toxicity ranking in the following order: AMP-CZO > AMP > CZO. The difference in the 96 h toxicity of AMP and CZO to algae may come as a result of the differences in their respective medicinal uses. The toxicity of *S. capricornutum* displayed concentration-dependent behaviors under exposure to AMP, CZO, and AMP-CZO; higher concentrations caused higher levels of inhibition. The interaction type of the AMP-CZO mixture is closely related to the components and subjects, as well as the exposure dose and mixing ratio.

AMP, CZO, and their mixtures had significant effects on the algae’s antioxidant system. AMP treatment reduces soluble protein content, increasing GSH, SOD, and CAT activities, and raising MDA levels, which is indicative of oxidative stress. The CZO treatment group had an effect on the antioxidant and photosynthetic systems of algae, and the highest growth point aligned with the minimum TP content. In low concentrations, AMP-CZO mixtures exhibited declining growth inhibition with rising treatment concentrations, accompanied by an increased photosynthetic pigment content. According to the MDR model, the relative inhibition rate of chlorophyll a in algae had a synergistic effect, which strengthened the inhibition of chlorophyll a in algae exposed to the AMP-CZO mixture, and hindered photosynthesis. At the same time, SOD indicators also showed a similar synergistic effect, disrupting the balance of the antioxidant system.

Differential gene expression analysis exposed numerous DEGs in AMP, CZO, and AMP-CZO exposures, which can be linked to vital biological processes. The suppression of algal growth observed in response to high-level AMP treatment can be linked to the inhibition of various signaling pathways related to xenobiotic metabolism and transportation, ribosome function, amino acid metabolism, nitrogen metabolism, and photosynthesis systems. SNARE interactions were observed in vesicular transport, ABC transporters, starch and sucrose metabolism, peroxisomes, and carotenoid biosynthesis in CZO; and plant–pathogen interactions, glycolysis/gluconeogenesis, cysteine and methionine metabolism, and fructose and mannose metabolism in AMP-CZO. In general, the synergy of the AMP-CZO mixture was mainly due to the down-regulation of *LHCA4*, *LHCA1*, *LHCA5*, and *sodA* genes, which affected photosynthesis and damaged the antioxidant system. In conclusion, our study sheds light on the ecological impact of AMP, CZO, and the mixture of the two in aquatic ecosystems, emphasizing the significance of comprehensive research in environmental risk assessments and in maintaining healthy aquatic ecosystems.

## Figures and Tables

**Table 1:** Antioxidant index value.

	Concentration (mol/L)	Growth Inhibition (%)	MDA ± Error (%)	GSH ± Error (%)	TP ± Error (%)	CAT ± Error (%)	SOD ± Error (%)	Chla ± Error (%)	Chlb ± Error (%)	Car ± Error (%)
AMP	3.46 × 10^−7^	32.57 ± 3.22	−1.79 ± 2.90	−3.11 ± 3.75	2.64 ± 3.05	−4.79 ± 1.04	−32.35 ± 8.82	71.56 ± 0.92	90.14 ± 4.82	47.32 ± 1.72
CZO	1.37 × 10^−6^	30.31 ± 4.03	−30.55 ± 6.23	−5.20 ± 7.14	36.92 ± 5.33	−14.25 ± 1.64	−7.46 ± 5.17	74.27 ± 1.46	37.41 ± 7.08	15.46 ± 4.18
AMP-CZO	1.71 × 10^−6^	38.23 ± 4.11	−7.23 ± 5.97	12.26 ± 5.52	−28.57 ± 7.80	−35.42 ± 9.55	−4.76 ± 8.20	74.40 ± 0.97	56.10 ± 1.16	50.22 ± 1.88

Note: The rejection rate of each index is ± error.

**Table 2:** The assembly statistics results.

Genes Number	GC Percentage	N50 Number	N50 Length	Max Length	Min Length	Average Length	Total Assembled Bases
24,660	69.59%	4753 bp	1970 bp	17,012 bp	201 bp	1211 bp	29,875,478

**Table 3:** Rate statistics comparison table.

Sample	Total	Unmapped (%)	Unique-Mapped (%)	Multiple-Mapped (%)	Total-Mapped (%)
AMP-1	41,551,672	6,109,037 (14.70%)	35,047,737 (84.35%)	394,898 (0.95%)	35,442,635 (85.30%)
AMP-2	35,883,734	5,220,400 (14.55%)	30,321,356 (84.50%)	341,978 (0.95%)	30,663,334 (85.45%)
AMP-3	37,367,704	5,482,929 (14.67%)	31,526,708 (84.37%)	358,067 (0.96%)	31,884,775 (85.33%)
AMP-CZO-1	41,117,304	5,948,503 (14.47%)	34,788,501 (84.61%)	380,300 (0.92%)	35,168,801 (85.53%)
AMP-CZO-2	37,209,628	5,368,445 (14.43%)	31,493,773 (84.64%)	347,410 (0.93%)	31,841,183 (85.57%)
AMP-CZO-3	54,201,918	7,947,135 (14.66%)	45,745,313 (84.40%)	509,470 (0.94%)	46,254,783 (85.34%)
CK-1	41,323,488	6,088,175 (14.73%)	34,821,109 (84.26%)	414,204 (1.00%)	35,235,313 (85.27%)
CK-2	39,380,936	5,647,707 (14.34%)	33,342,932 (84.67%)	390,297 (0.99%)	33,733,229 (85.66%)
CK-3	40,895,384	6,079,715 (14.87%)	34,416,111 (84.16%)	399,558 (0.98%)	34,815,669 (85.13%)
CZO-1	37,145,804	5,346,149 (14.39%)	31,450,299 (84.67%)	349,356 (0.94%)	31,799,655 (85.61%)
CZO-2	36,636,374	5,257,166 (14.35%)	31,032,882 (84.71%)	346,326 (0.95%)	31,379,208 (85.65%)
CZO-3	40,705,028	6,101,978 (14.99%)	34,223,736 (84.08%)	379,314 (0.93%)	34,603,050 (85.01%)

**Table 4:** KEGG pathways significantly enriched.

Category	Pathway	*p* Value	Pathway ID	Up-Regulated Genes	Down-Regulated Genes
Control vs. AMP					
Energy metabolism	Nitrogen metabolism	0.0080	ko00910	CYP55	NR, NRT, glnA
Carbohydrate metabolism	Starch and sucrose metabolism	0.0097	ko00500		
Energy metabolism	Photosynthesis—antenna proteins	0.016	ko00196	LCHA2, LCHB4	LCHA1, LCHA4
Signal transduction	Plant hormone signal transduction	0.041	ko04075	IAA	PP2C
Control vs. CZO					
Lipid metabolism	Fatty acid elongation	1.37 × 10^−5^	ko00062		
Folding, sorting, and degradation	SNARE interactions in vesicular transport	2.68 × 10^−4^	ko04130		SYP7, SEC22, STX5, SFT1, STX4, GOSR1, GOSR2
Membrane transport	ABC transporters	4.46 × 10^−4^	ko02010		ABCG2, ABCG12, ABCG22, ABCC2
Lipid metabolism	Cutin, suberine, and wax biosynthesis	6.83 × 10^−4^	ko00073		CYP94A5, FAR
Environmental adaptation	Plant–pathogen interaction	0.00121	ko04626		KCS1, KSC2, KCS3, KCS4, KCS5, KCS6, KCS7, KCS8, KCS9, KCS10
Signal transduction	MAPK signaling pathway—plant	0.00125	ko04016		
Carbohydrate metabolism	Starch and sucrose metabolism	0.0117	ko00500		
Replication and repair	DNA replication	0.0165	ko03030		
Carbohydrate metabolism	Ascorbate and aldarate metabolism	0.0235	ko00053		
Glycan biosynthesis and metabolism	Other types of O-glycan biosynthesis	0.0312	ko00514		
Transport and catabolism	Peroxisome	0.0376	ko04146		GPX1, sodA
Biosynthesis of other secondary metabolites	Anthocyanin biosynthesis	0.0415	ko00942		
Metabolism of terpenoids and polyketides	Carotenoid biosynthesis	0.0461	ko00906		LCY1
Control vs. AMP-CZO					
Lipid metabolism	Fatty acid elongation	6.97 × 10^−7^	ko00062		
Environmental adaptation	Plant–pathogen interaction	1.35 × 10^−4^	ko04626		
Membrane transport	ABC transporters	5.01 × 10^−4^	ko02010	ABCC2	ABCG52, Abcg2, ABCG22,
Carbohydrate metabolism	Glycolysis/Gluconeogenesis	0.0018	ko00010		
Global and overview maps	Metabolic pathways	0.0018	ko01100		LHCA4, PSAK, PSAE
Replication and repair	DNA replication	0.0054	ko03030		
Replication and repair	Base excision repair	0.0057	ko03410		
Global and overview maps	Biosynthesis of secondary metabolites	0.0099	ko01110		Prp4, got1, asp5, glt1, E2.6.1.42
Amino acid metabolism	Cysteine and methionine metabolism	0.0115	ko00270		
Carbohydrate metabolism	Ascorbate and aldarate metabolism	0.0203	ko00053		
Biosynthesis of other secondary metabolites	Anthocyanin biosynthesis	0.0229	ko00942		
Lipid metabolism	Cutin, suberine, and wax biosynthesis	0.0230	ko00073		
Amino acid metabolism	Lysine degradation	0.0388	ko00310		
Carbohydrate metabolism	Fructose and mannose metabolism	0.0391	ko00051		

## Data Availability

Data is contained within the article or Supplementary Material.

## Supplementary Materials

The following supporting information can be downloaded at: https://www.mdpi.com/article/10.3390/toxics12030217/s1, Figure S1: Testing and analysis process; Figure S2: Four major database annotations Venn diagrams, Figure S3: GO secondary classification chart; Figure S4: (A) Sample principal component analysis; (B) Sample correlation heatmap; Figure S5: GO terms; Figure S6 KEGG pathway.

## References

[1] Gomaa M., Zien-Elabdeen A., Hifney A.F., Adam M.S. (2021). Phycotoxicity of antibiotics and non-steroidal anti-inflammatory drugs to green algae *Chlorella* sp. and *Desmodesmus spinosus*: Assessment of combined toxicity by Box–Behnken experimental design. Environ. Technol. Innov..

[2] Aydogdu S., Hatipoglu A. (2023). Theoretical insights into the reaction mechanism and kinetics of ampicillin degradation with hydroxyl radical. J. Mol. Model..

[3] Gavrilescu M., Demnerová K., Aamand J., Agathos S., Fava F. (2015). Emerging pollutants in the environment: Present and future challenges in biomonitoring, ecological risks and bioremediation. New Biotechnol..

[4] Gu J.D., Wang Y.S. (2015). Coastal and marine pollution and ecotoxicology. Ecotoxicology.

[5] Fang T.H., Nan F.H., Chin T.S., Feng H.M. (2012). The occurrence and distribution of pharmaceutical compounds in the effluents of a major sewage treatment plant in Northern Taiwan and the receiving coastal waters. Mar. Pollut. Bull..

[6] Liu S., Zhang J., Zhang Y., Qin L. (2012). APTox: Assessment and prediction of toxicity of chemical mixtures. J. Chem..

[7] Zhong Q.-L., Chen Z., Shen Q., Xiong J.-Q. (2023). Occurrence of antibiotics in reclaimed water, and their uptake dynamics, phytotoxicity, and metabolic fate in *Lolium perenne* L.. Sci. Total Environ..

[8] Feng L., Xu H., Wang Y. (2020). Study of the effects of three β-lactam antibiotics on large toxicity. Environ. Sci. Technol..

[9] Wang C., He M., Wu C., Chen Z., Jiang L., Wang C. (2023). Toxicity interaction of polystyrene nanoplastics with sulfamethoxazole on the microalgae *Chlamydomonas reinhardtii*: A closer look at effect of light availability. J. Environ. Manag..

[10] Xie H., Hao H., Xu N., Liang X., Gao D., Xu Y., Gao Y., Tao H., Wong M. (2019). Pharmaceuticals and personal care products in water, sediments, aquatic organisms, and fish feeds in the pearl river delta: Occurrence, distribution, potential sources, and health risk assessment. Sci. Total Environ..

[11] Chia M.A., Lorenzi A.S., Ameh I., Dauda S., Cordeiro-Araújo M.K., Agee J.T., Okpanachi I.Y., Adesalu A.T. (2021). Susceptibility of phytoplankton to the increasing presence of active pharmaceutical ingredients (APIs) in the aquatic environment: A review. Aquat. Toxicol..

[12] Mo J., Lv R., Qin X., Wu X., Chen H., Yan N., Shi J., Wu Y., Liu W., Kong R.Y.C. (2023). Mechanistic insights into hormesis induced by erythromycin in the marine alga *Thalassiosira weissflogii*. Ecotoxicol. Environ. Saf..

[13] Zhang Y., He D., Chang F., Dang C., Fu J. (2021). Combined effects of sulfamethoxazole and erythromycin on a freshwater microalga, *Raphidocelis subcapitata*: Toxicity and oxidative stress. Antibiotics.

[14] Li M., Zhou H., Ye M., Xu X., Pang L., Zhao Z., Xuan Y. (2023). Interactions between typical antibiotics and *Microcystis aeruginosa* in aquatic environment. Clean Soil. Air Water.

[15] Petrie B., Barden R., Kasprzyk-Hordern B. (2015). A review on emerging contaminants in wastewaters and the environment: Current knowledge, understudied areas and recommendations for future monitoring. Water Res..

[16] Zhong X., Zhu Y., Wang Y., Zhao Q., Huang H. (2021). Effects of three antibiotics on growth and antioxidant response of *Chlorella pyrenoidosa* and *Anabaena cylindrica*. Ecotoxicol. Environ. Saf..

[17] Rozas O., Contreras D., Mondaca M.A., Pérez-Moya M., Mansilla H.D. (2010). Experimental design of Fenton and photo-Fenton reactions for the treatment of ampicillin solutions. J. Hazard. Mater..

[18] Shukla A., Khan E., Srivastava A., Tandon P., Sinha K. (2016). A computational study on molecular structure, multiple interactions, chemical reactivity and molecular docking studies on 6[D (−) α-amino-phenylacetamido] penicillanic acid (ampicillin). Mol. Simul..

[19] Zhang Q., Demeestere K., De Schamphelaere K.A.C. (2023). The influence of pH and dissolved organic carbon on the ecotoxicity of ampicillin and clarithromycin. Sci. Total Environ..

[20] Loannou-Ttofa L., Raj S., Prakash H., Fatta-Kassinos D. (2019). Solar photo-Fenton oxidation for the removal of ampicillin, total cultivable and resistant *E. coli* and ecotoxicity from secondary-treated wastewater efuents. Chem. Eng. J..

[21] Singh V., Pandey B., Suthar S. (2018). Phytotoxicity of amoxicillin to the duckweed *Spirodela polyrhiza*: Growth, oxidative stress, biochemical traits and antibiotic degradation. Chemosphere.

[22] Nie X. (2017). Multiple β-Lactam Antibiotics in Water Based on LC-M S/MS.

[23] Diwan V., Tamhankar A.J., Aggarwal M., Sen S., Khandal R.K., Lundborg C.S. (2009). Detection of antibiotics in hospital effluents in India. Curr. Sci..

[24] Chen H., Li X., Zhu S. (2012). Occurrence and distribution of selected pharmaceuticals and personal care products in aquatic environments: A comparative study of regions in China with different urbanization levels. Environ. Sci. Pollut. Res..

[25] Jia L. (2012). Distribution and Transfer and Transformation of β-Lactam Antibiotics in Sewage Treatment Plants.

[26] Anderson P.D., D’Aco V.J., Shanahan P., Chapra S.C., Buzby M.E., Cunningham V.L., DuPlessie B.M., Hayes E.P., Mastrocco F.J., Parke N.J. (2004). Screening analysis of human pharmaceutical compounds in US surface waters. Environ. Sci. Technol..

[27] Chen Y., Xu D.Q. (2010). Two patterns of leaf photosynthetic response to irradiance transition from saturating to limiting one in some plant species. New Phytol..

[28] Koussevitzky S., Nott A., Mockler T.C., Hong F., Sachetto-Martins C., Surpin M., Lim J., Mittler R., Chory J. (2007). Signals from chloroplasts converge to regulate nuclear gene expression. Science.

[29] Mallick N., Mohn F.H. (2000). Reactive oxygen species: Response of algal cells. J. Plant Physiol..

[30] Xue X., Su X., Zhou L., Ji J., Qin Z., Liu J., Li K., Wang H., Wang Z. (2023). Antibiotic-Induced Recruitment of Specific Algae-Associated Microbiome Enhances the Adaptability of *Chlorella vulgaris* to Antibiotic Stress and Incidence of Antibiotic Resistance. Environ. Sci. Technol..

[31] Liu Y., Yue L., Wang C., Zhu X., Wang Z., Xing B. (2020). Photosynthetic response mechanisms in typical C3 and C4 plants upon La_2_O_3_ nanoparticle exposure. Environ. Sci. Nano.

[32] Qian H., Pan X., Chen J., Zhou D., Chen Z., Zhang L., Fu Z. (2012). Analyses of gene expression and physiological changes in *Microcystis aeruginosa* reveal the phytotoxicities of three environmental pollutants. Ecotoxicology.

[33] Lindberg J., Lundeberg J. (2010). The plasticity of the mammalian transcriptome. Genomics.

[34] Eguchi K., Nagase H., Ozawa M., Endoh Y.S., Goto K., Hirata K., Miyamoto K., Yoshimura H. (2004). Evaluation of antimicrobial agents for veterinary use in the ecotoxicity test using microalgae. Chemosphere.

[35] Enick O.V., Moore M.M. (2007). Assessing the assessments: Pharmaceuticals in the environment. Environ. Impact Assess. Rev..

[36] Huang F.-L., Liu M., Qin L.-T., Mo L., Liang Y., Zeng H., Deng Z. (2023). Toxicity interactions of azole fungicide mixtures on *Chlorella pyrenoidosa*. Environ. Toxicol..

[37] Li B., Zhang T., Xu Z., Fang H.H.P. (2009). Rapid analysis of 21 antibiotics of multiple classes in municipal wastewater using ultra performance liquid chromatography–tandem mass spectrometry. Anal. Chim. Acta.

[38] Berenbaum M.C. (1989). What is synergy?. Pharmacol. Rev..

[39] Georgiou C.D., Grintzalis K., Zervoudakis G., Papapostolou I. (2008). Mechanism of coomassie brilliant blue g-250 binding to proteins: A hydrophobic assay for nanogram quantities of proteins. Anal. Bioanal. Chem..

[40] Angel B.M., Vallotton P., Apte S.C. (2015). On the mechanism of nanoparticulate CeO_2_ toxicity to freshwater algae. Aquat. Toxicol..

[41] Liu B. (2011). Toxic Effects of Erythromycin, Ciprofloxacin, and Sulfamethoxazole on Sheep Horn Crescent Algae and Their Mechanism of Action. Environmental Pollution.

[42] Noctor G., De Paepe R., Foyer C.H. (2007). Mitochondrial redox biology and homeostasis in plants. Trends Plant Sci..

[43] Zhang Y.-H., Liu S.-S., Song X.-Q., Ge H.-L. (2008). Prediction for the mixture toxicity of six organophosphorus pesticides to the luminescent bacterium Q67. Ecotoxicol. Environ. Saf..

[44] Liu S.-S., Song X.-Q., Liu H.-L., Zhang Y.-H., Zhang J. (2009). Combined photobacterium toxicity of herbicide mixtures containing one insecticide. Chemosphere.

[45] Chen S.G., Yang J., Zhang M.S., Strasser R.J., Qiang S. (2016). Classification and characteristics of heat tolerance in *Ageratina adenophora* populations using fast chlorophyll a fluorescence rise O-J-I-P. Environ. Exp. Bot..

[46] Zhang R., Yin J., Sui Z., Han L., Li Y., Huang J. (2022). Biocontrol of antifungal volatiles produced by *Ceriporia lacerate* HG2011 against citrus fruit rot incited by *Penicillium* spp.. Postharvest Biol. Technol..

[47] Guo J., Selby K., Boxall A.B. (2016). Comparing the sensitivity of chlorophytes, cyanobacteria, and diatoms to major-use antibiotics. Environ. Toxicol. Chem..

[48] Qin L.-T., Liu S.-S., Zhang J., Xiao Q.-F. (2011). A novel model integrated concentration addition with independent action for the prediction of toxicity of multi-component mixture. Toxicology.

[49] Bliss C.I. (1939). The toxicity of poisons applied jointly. Ann. Appl. Biol..

[50] Belden J.B., Gilliom R.J., Lydy M.J. (2007). How Well Can We Predict the Toxicity of Pesticide Mixtures to Aquatic Life?. Integr. Environ. Assess. Manag..

[51] Cedergreen N. (2014). Quantifying synergy a systematic review of mixture toxicity studies within environmental toxicology. PLoS ONE.

[52] Bradford M.M. (1976). A rapid and sensitive method for the quantitation of microgram quantities of protein utilizing the principle of protein-dye binding. Anal. Biochem..

[53] Aebi H. (1984). Catalase in vitro. Methods Enzymol..

[54] Charles B., Irwin F. (1971). Superoxide dismutase: Improved assays and an assay applicable to acrylamide gels. Anal. Biochem..

[55] Gonzalez-Pleiter M., Gonzalo S., Rodea-Palomares I., Leganes F., Rosal R., Boltes K., Marco E., Fernandez-Pinas F. (2013). Toxicity of five antibiotics and their mixtures towards photosynthetic aquatic organisms: Implications for environmental risk assessment. Water Res..

[56] Wu Y., Wan L., Zhang W., Ding H., Yang W. (2020). Resistance of cyanobacteria *Microcystis aeruginosa* to erythromycin with multiple exposure. Chemosphere.

[57] Hedlund E., Deng Q. (2018). Single-cell RNA sequencing: Technical advancements and biological applications. Mol. Asp. Med..

[58] Cha J., Yang S., Carlson K.H. (2015). Occurrence of β-lactam and polyether ionophore antibiotics in surface water, urban wastewater, and sediment. Geosystem Eng..

[59] Backhaus T. (2009). Environmental Risk Assessment of Pharmaceutical Mixtures: Demands, Gaps and Possible Bridges. AAPS J..

[60] Backhaus T., Altenburger R., Boedeker W., Faust M., Scholze M., Grimme L.H. (2009). Predictability of the toxicity of a multiple mixture of dissimilarly acting chemicals to *Vibrio fischeri*. Environ. Toxicol. Chem..

[61] Magdaleno A., Saenz M.E., Juárez A.B., Moretton J. (2015). Effects of six antibiotics and their binary mixtures on growth of *Pseudokirchneriella subcapitata*. Ecotoxicol. Environ. Saf..

[62] Carusso S., Juárez A.B., Moretton J., Magdaleno A. (2018). Effects of three veterinary antibiotics and their binary mixtures on two green alga species. Chemosphere.

[63] Białk-Bielińska A., Caban M., Pieczyńska A., Stepnowski P., Stolte S. (2017). Mixture toxicity of six sulfonamides and their two transformation products to green algae *Scenedesmus vacuolatus* and duckweed *Lemna minor*. Chemosphere.

[64] Teixeira J.R., Granek E.F. (2017). Effects of environmentally-relevant antibiotic mixtures on marine microalgal growth. Sci. Total Environ..

[65] Evans R.M., Martin O.V., Faust M., Kortenkamp A. (2016). Should the scope of human mixture risk assessment span legislative/regulatory silos for chemicals?. Sci. Total Environ..

[66] Syberg K., Elleby A., Pedersen H., Cedergreen N., Forbes V.E. (2008). Mixture toxicity of three toxicants with similar and dissimilar modes of action to *Daphnia magna*. Ecotoxicol. Environ. Saf..

[67] Tang J., Fang J., Tam N.F., Yang Y., Dai Y., Zhang J., Shi Y. (2021). Impact of Phytoplankton Blooms on concentrations of antibiotics in sediment and snails in a subtropical river, China. Environ. Sci. Technol..

[68] Zhang M., Steinman A.D., Xue Q., Zhao Y., Xu Y., Xie L. (2020). Effects of erythromycin and sulfamethoxazole on *Microcystis aeruginosa*: Cytotoxic endpoints, production and release of microcystin-LR. J. Hazard. Mater..

[69] Wang Z., Chen Q., Zhang J., Dong J., Ao Y., Wang M., Wang X. (2019). Long-term exposure to antibiotic mixtures favors microcystin synthesis and release in *Microcystis aeruginosa* with different morphologies. Chemosphere.

[70] Jiang Y., Liu Y., Zhang J. (2020). Antibiotic contaminants reduced the treatment efficiency of UV-C on Microcystis aeruginosa through hormesis. Environ. Pollut..

[71] Jiang Y., Liu Y., Zhang J. (2021). Mechanisms for the stimulatory effects of a five-component mixture of antibiotics in *Microcystis aeruginosa* at transcriptomic and proteomic levels. J. Hazard. Mater..

[72] Wang Z., Chen Q., Hu L., Wang M. (2018). Combined effects of binary antibiotic mixture on growth, microcystin production, and extracellular release of Microcystis aeruginosa: Application of response surface methodology. Environ. Sci. Pollut. Res..

[73] Liu Y., Chen S., Zhang J., Li X., Gao B. (2017). Stimulation effects of ciprofloxacin and sulphamethoxazole in *Microcystis aeruginosa* and isobaric tag for relative and absolute quantitation-based screening of antibiotic targets. Mol. Ecol..

[74] Xu S., Liu Y., Zhang J., Gao B. (2021). Proteomic mechanisms for the combined stimulatory effects of glyphosate and antibiotic contaminants on *Microcystis aeruginosa*. Chemosphere.

[75] Chaput V., Martin A., Lejay L. (2020). Redox metabolism: The hidden player in carbon and nitrogen signaling?. J. Exp. Bot..

[76] Smith S.R., Dupont C.L., McCarthy J.K., Broddrick J.T., Oborník M., Horák A., Allen A.E. (2019). Evolution and regulation of nitrogen flux through compartmentalized metabolic networks in a marine diatom. Nat. Commun..

[77] Cho Y.-H., Yoo S.-D. (2011). Signaling Role of Fructose Mediated by FINS1/FBP in *Arabidopsis thaliana*. PLoS Genet..

[78] Rolland F., Baena-Gonzalez E., Sheen J. (2006). Sugar Sensing and Signaling in Plants: Conserved and Novel Mechanisms. Ann. Rev. Plant Biol..

[79] Ende W.V.D., Valluru R. (2008). Sucrose, sucrosyl oligosaccharides, and oxidative stress: Scavenging and salvaging?. J. Exp. Bot..

[80] Furlan A., Llanes A., Luna V., Castro S. (2012). Physiological and Biochemical Responses to Drought Stress and Subsequent Rehydration in the Symbiotic Association Peanut-*Bradyrhizobium* sp.. ISRN Agron..

[81] Mortimer M., Kasemets K., Vodovnik M., Marinšek-Logar R., Kahru A. (2011). Exposure to CuO Nanoparticles Changes the Fatty Acid Composition of Protozoa *Tetrahymena thermophila*. Environ. Sci. Technol..

[82] Hernandez-Marin E., Martínez A. (2012). Carbohydrates and Their Free Radical Scavenging Capability: A Theoretical Study. J. Phys. Chem. B.

[83] Li L., Zhang L., Gong F., Liu J. (2020). Transcriptomic analysis of hydrogen photoproduction in *Chlorella pyrenoidosa* under nitrogen deprivation. Algal Res..

[84] Wang P., Grimm B. (2021). Connecting chlorophyll metabolism with accumulation of the photosynthetic apparatus. Trends Plant Sci..

[85] Fan G.D., Zhou J.J., Zheng X.M., Chen W. (2018). Growth inhibition of *Microcystis aeruginosa* by copper-based MOFs: Performance and physiological effect on algal cells. Appl. Organomet. Chem..

[86] Li Y., Liu X., Zheng X., Yang M., Gao X., Huang J., Zhang L., Fan Z. (2020). Toxic effects and mechanisms of PFOA and its substitute GenX on the photosynthesis of *Chlorella pyrenoidosa*. Sci. Total Environ..

[87] Liu K., Zhang D., Xiao X., Cui L., Zhang H. (2020). Occurrence of quinotone antibiotics and their impacts on aquatic environment in typical riverestuary system of Jiaozhou Bay, China. Ecotoxicol. Environ. Saf..

[88] Moller I.M. (2001). Plant Mitochondria and Oxidative Stress: Electron transport, NADPH turnover, and metabolism of reactive oxygen species. Ann. Rev. Plant Physiol. Plant Mol. Biol..

[89] Kanerva M., Routti H., Tamuz Y., Nyman M., Nikinmaa M. (2012). Antioxidative defense and oxidative stress in ringed seals (*Pusa hispida*) from differently polluted areas. Aquat. Toxicol..

[90] Zou W., Zhou Q., Zhang X., Hu X. (2018). Environmental transformations and algal toxicity of single-layer molybdenum disulfide regulated by humic acid. Environ. Sci. Technol..

[91] Zhao X., Han B.D., Li L.X. (2012). Function of SM protein in vesicle transport. Hereditas.

[92] Wang T., Li L., Hong W. (2017). SNARE proteins in membrane trafficking. Traffic.

[93] Gaggar P., Kumar M., Mukhopadhyay K. (2021). Genome-scale identification, in silico characterization and interaction study between wheat SNARE and NPSN gene families involved in vesicular transport. IEEE/ACM Trans. Comput. Biol. Bioinform..

[94] Andreeva A.V., Kutuzov M.A., Evans D.E., Hawes C.R. (1998). The structure and function of the Golgi apparatus: A hundred years of questions. J. Exp. Bot..

[95] Saftig P., Klumperman J. (2009). Lysosome biogenesis and lysosomal membrane proteins: Trafficking meets function. Nat. Rev. Mol. Cell Biol..

[96] Gindhart J.G., Weber K.P. (2009). Lysosome and endosome organization and transport in neurons. Encycl. Neurosci..

[97] Rodriguez-Concepcion M., Avalos J., Bonet M.L., Boronat A., Gomez-Gomez L., Hornero-Mendez D., Zhu C. (2018). A global perspective on carotenoids: Metabolism, biotechnology, and benefits for nutrition and health. Prog. Lipid Res..

[98] Jahns P., Holzwarth A.R. (2012). The role of the xanthophyll cycle and of lutein in photoprotection of photosystem II. Biochim. Biophys. Acta.

[99] Widomska J., Welc R., Gruszecki W.I. (2019). The effect of carotenoids on the concentration of singlet oxygen in lipid membranes. Biochim. Biophys. Acta Biomembr..

[100] Britton G., Liaaen-Jensen S., Pfander H. (1998). Carotenoids Volume 3: Biosynthesis and Metabolism.

[101] Britton G., Liaaen-Jensen S., Pfander H. (2008). Carotenoids Volume 4: Natural Functions.

[102] Causin H.F. (1996). The central role of amino acids on nitrogen utilization and plant growth. J. Plant Physiol..

[103] Kumar A., Bera S. (2020). Revisiting nitrogen utilization in algae: A review on the process of regulation and assimilation. Bioresour. Technol. Rep..

[104] Martínez-Lüscher J., Torres N., Hilbert G., Richard T., Sánchez-Díaz M., Delrot S., Aguirreolea J., Pascual I., Gomès E. (1998). Ultraviolet-B radiation modifies the quantitative and qualitative profile of flavonoids and amino acids in grape berries. Phytochemistry.

[105] Yue M., Li Y., Wang X. (1998). Effects of enhanced ultraviolet-B radiation on plant nutrients and decomposition of spring wheat under field conditions. Environ. Exp. Bot..

[106] Ashraf M., Foolad M. (2007). Roles of glycine betaine and proline in improving plant abiotic stress resistance. Environ. Exp. Bot..

[107] Wu Z., Liu S., Zhao J., Wang F., Du Y., Zou S., Li H., Wen D., Huang Y. (2017). Comparative responses to silicon and selenium in relation to antioxidant enzyme system and the glutathione-ascorbate cycle in flowering Chinese cabbage (*Brassica campestris* L. ssp. *chinensis* var. *utilis*) under cadmium stress. Environ. Exp. Bot..

[108] Pikula K.S., Zakharenko A.M., Chaika V.V., Stratidakis A.K., Kokkinakis M., Waissi G., Rakitskii V.N., Sarigiannis D.A., Hayes A.W., Coleman M.D. (2019). Toxicity bioassay of waste cooking oil-based biodiesel on marine microalgae. Toxicol. Rep..

[109] Wu Y.M., Guo P.Y., Zhang X.Y., Zhang Y.X., Xie S.T., Deng J. (2019). Effect of microplastics exposure on the photosynthesis system of freshwater algae. J. Hazard. Mater..

[110] Wan L., Wu Y., Ding H., Zhang W. (2020). Toxicity, biodegradation, and metabolic fate of organophosphorus pesticide trichlorfon on the freshwater algae chlamydomonas reinhardtii. J. Agri. Food. Chem..

[111] Gao G., Shi Q., Xu Z., Xu J., Campbell D.A., Wu H. (2018). Global warming interacts with ocean acidification to alter PSII function and protection in the diatom *Thalassiosira weissflogii*. Environ. Exp. Bot..

[112] Gao G., Xu Z., Shi Q., Wu H. (2018). Increased CO_2_ exacerbates the stress of ultraviolet radiation on photosystem II function in the diatom *Thalassiosira weissflogii*. Environ. Exp. Bot..

