# Supplementary material for: Mechanism of the Synergistic Toxicity of Ampicillin and Cefazoline on Selenastrum capricornutum
Source: Toxics. 2024 Mar 14;12(3):217. doi: 10.3390/toxics12030217 (PMC10974183; doi:10.3390/toxics12030217)
Supplement: Supplementary file 1 [file toxics-12-00217-s001.zip › toxics-2882501-supplementary.pdf]

Supporting Information

Synergistic mechanism of ampicillin and cefazoline on *Selenastrum capricornutum*

Feng-Ling Huang[a], Li-Tang Qin[a,b,c,*], Ling-Yun Mo[b,c,d], Hong-Hu Zeng[a,b,c], Yan-Peng Liang[a,b,c]

[a]College of Environment Science and Engineering, Guilin University of Technology, Guilin 541004, China

[b]Guangxi Key Laboratory of Environmental Pollution Control Theory and Technology, Guilin 541004, China

[c]Collaborative Innovation Center for Water Pollution Control and Water Safety in Karst Area, Guilin University of Technology, Guilin 541004, China

[d]Technical Innovation Center of Mine Geological Environmental Restoration Engineering in Southern Karst Area, Nanjing 530029, China

Contents

Figure S1 Testing and analysis process

Figure S2 Four major database annotations Venn diagrams

Figure S3 GO secondary classification chart

Figure S4A Sample principal component analysis

Figure S4B Sample correlation heatmap

Figure S5 GO terms

Figure S6 KEGG pathway

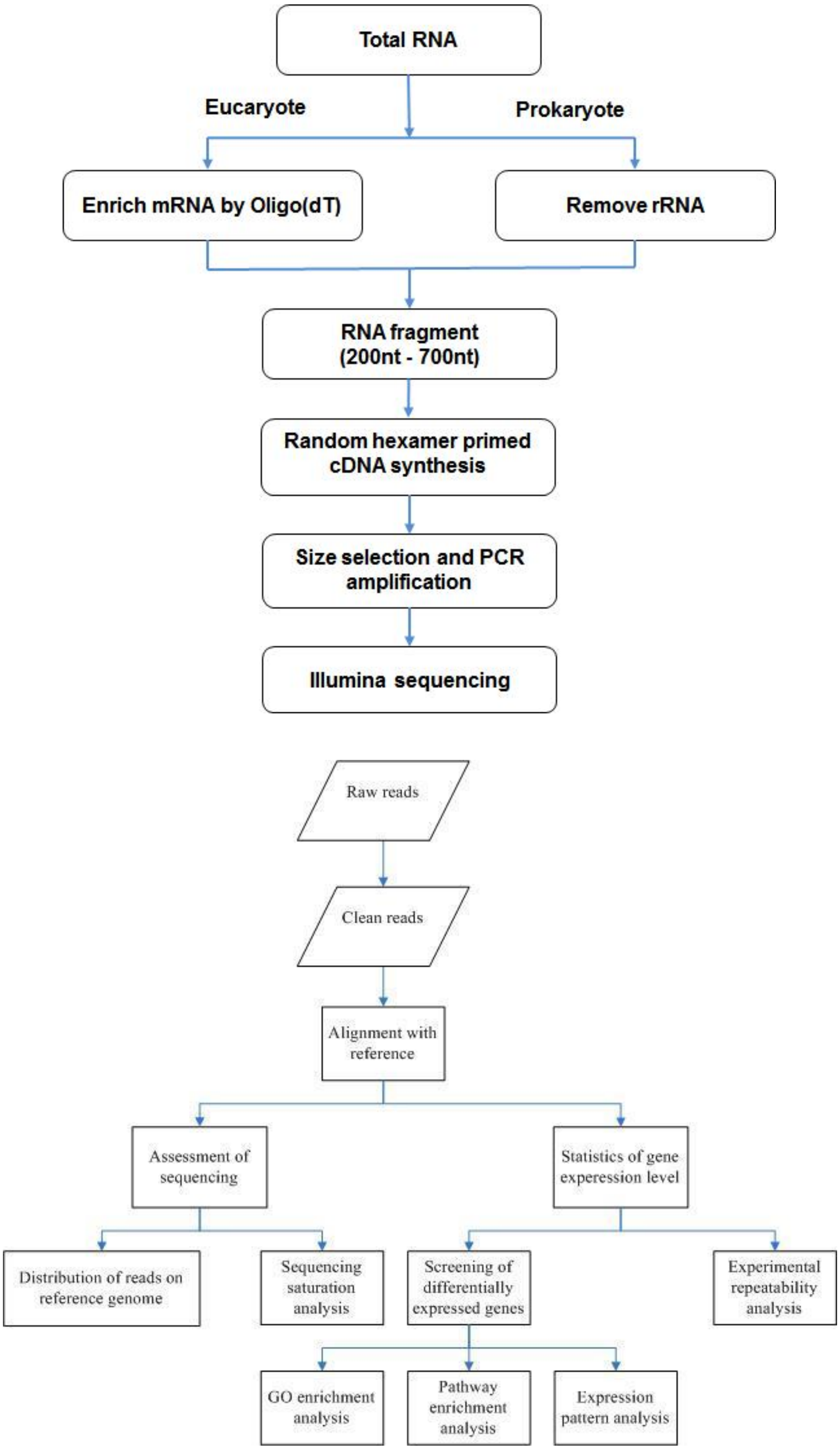

Figure S1 Testing and analysis process

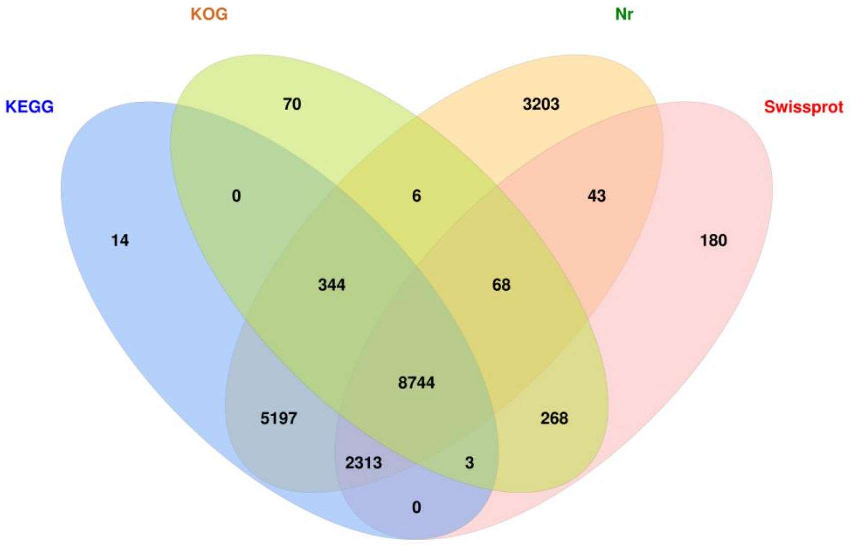

Figure S2 Four major database annotations Venn diagrams

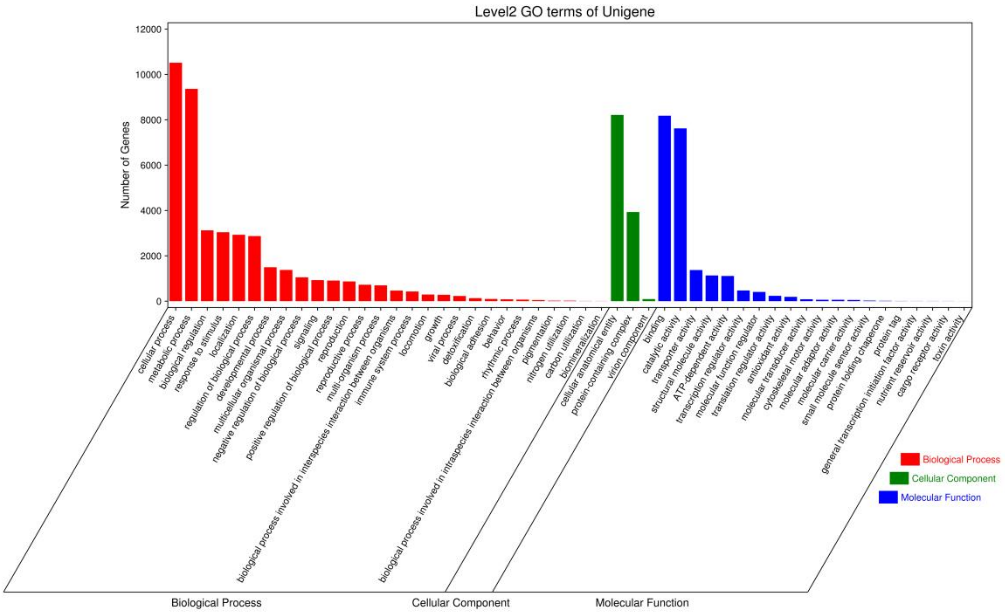

Figure S3 GO secondary classification chart

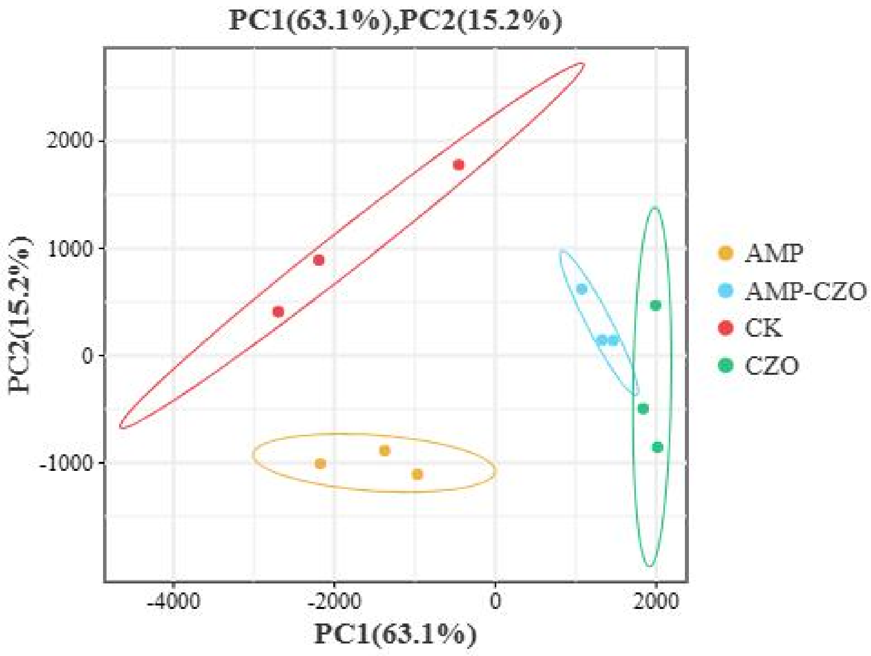

Figure S4A Sample principal component analysis

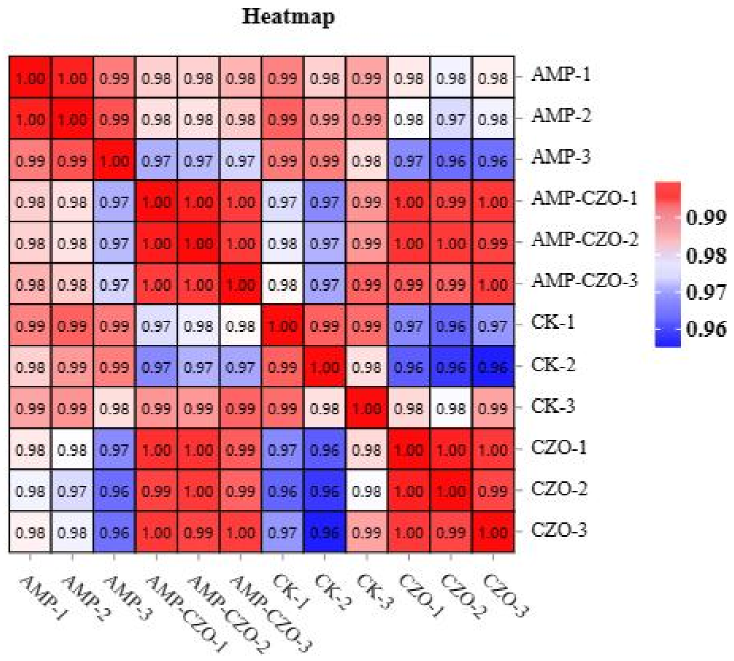

Figure S4B Sample correlation heatmap

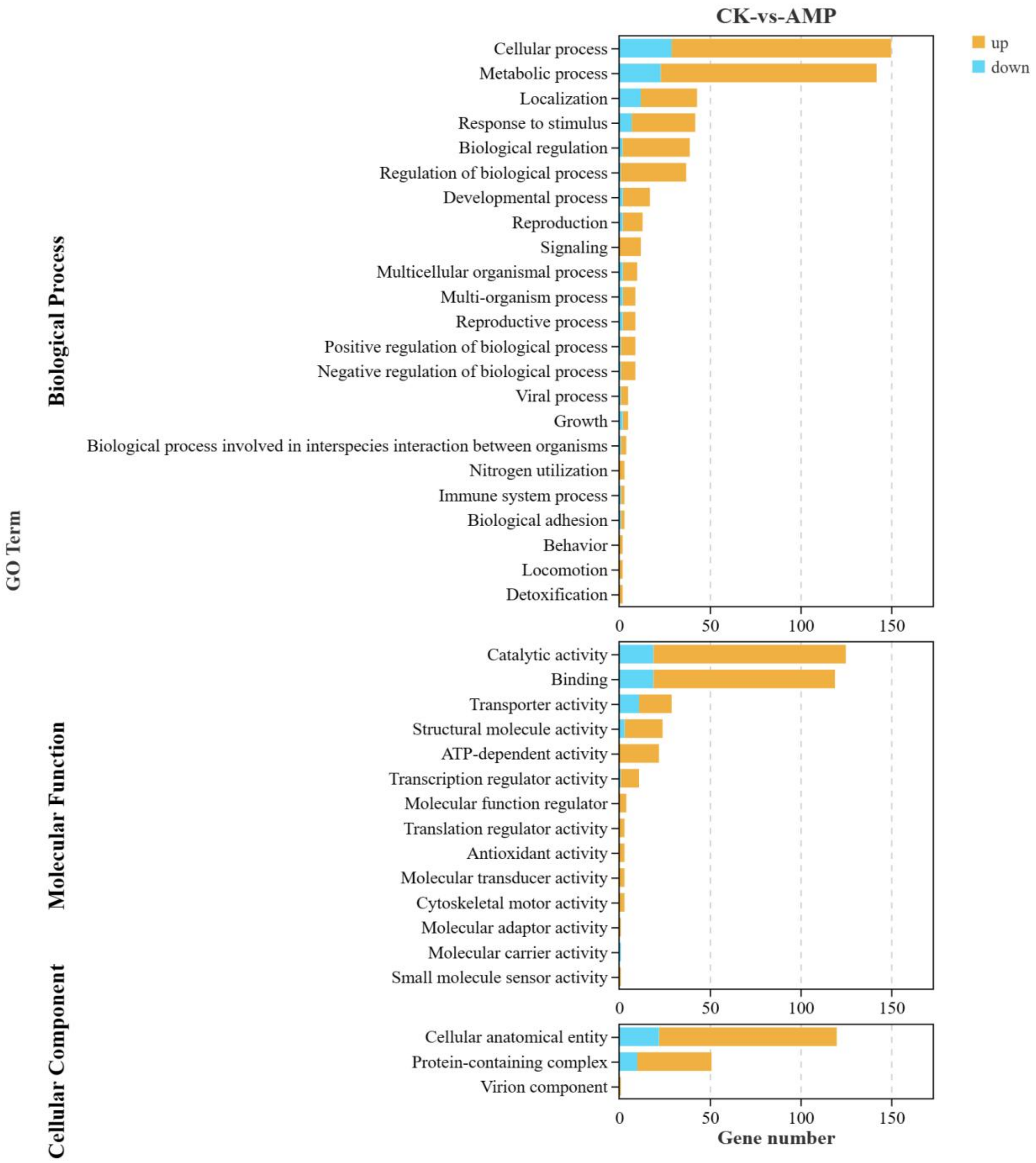

CK-vs-AMP
up
down
GO Term
Biological Process
Cellular process
Metabolic process
Localization
Response to stimulus
Biological regulation
Regulation of biological process
Developmental process
Reproduction
Signaling
Multicellular organismal process
Multi-organism process
Reproductive process
Positive regulation of biological process
Negative regulation of biological process
Viral process
Growth
Biological process involved in interspecies interaction between organisms
Nitrogen utilization
Immune system process
Biological adhesion
Behavior
Locomotion
Detoxification
Molecular Function
Catalytic activity
Binding
Transporter activity
Structural molecule activity
ATP-dependent activity
Transcription regulator activity
Molecular function regulator
Translation regulator activity
Antioxidant activity
Molecular transducer activity
Cytoskeletal motor activity
Molecular adaptor activity
Molecular carrier activity
Small molecule sensor activity
Cellular Component
Cellular anatomical entity
Protein-containing complex
Virion component
0
50
100
150
Gene number

A

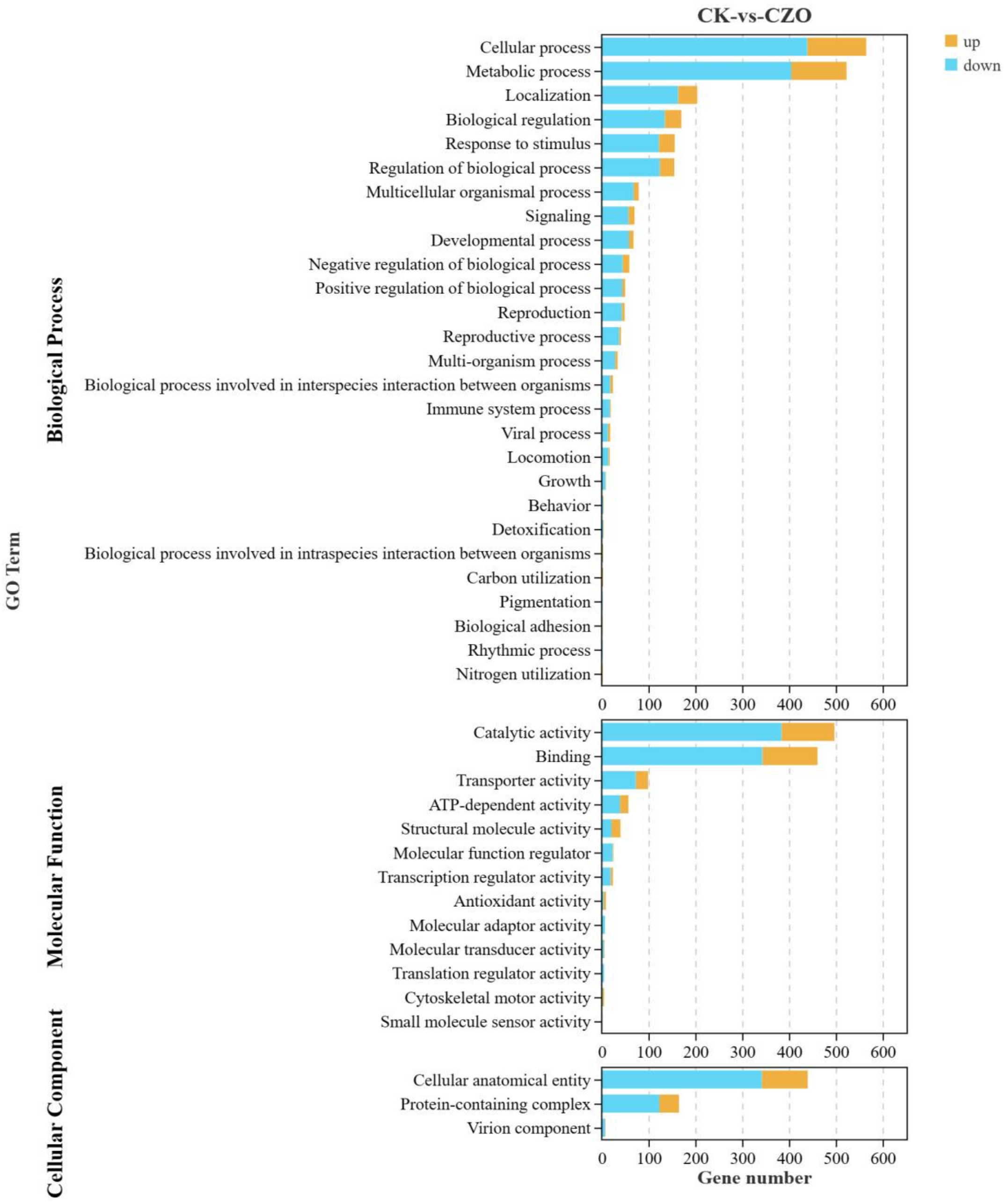
CK-vs-CZO
up
down
GO Term
Biological Process
Cellular process
Metabolic process
Localization
Biological regulation
Response to stimulus
Regulation of biological process
Multicellular organismal process
Signaling
Developmental process
Negative regulation of biological process
Positive regulation of biological process
Reproduction
Reproductive process
Multi-organism process
Biological process involved in interspecies interaction between organisms
Immune system process
Viral process
Locomotion
Growth
Behavior
Detoxification
Biological process involved in intraspecies interaction between organisms
Carbon utilization
Pigmentation
Biological adhesion
Rhythmic process
Nitrogen utilization
Molecular Function
Catalytic activity
Binding
Transporter activity
ATP-dependent activity
Structural molecule activity
Molecular function regulator
Transcription regulator activity
Antioxidant activity
Molecular adaptor activity
Molecular transducer activity
Translation regulator activity
Cytoskeletal motor activity
Small molecule sensor activity
Cellular Component
Cellular anatomical entity
Protein-containing complex
Virion component
0 100 200 300 400 500 600
Gene number

B

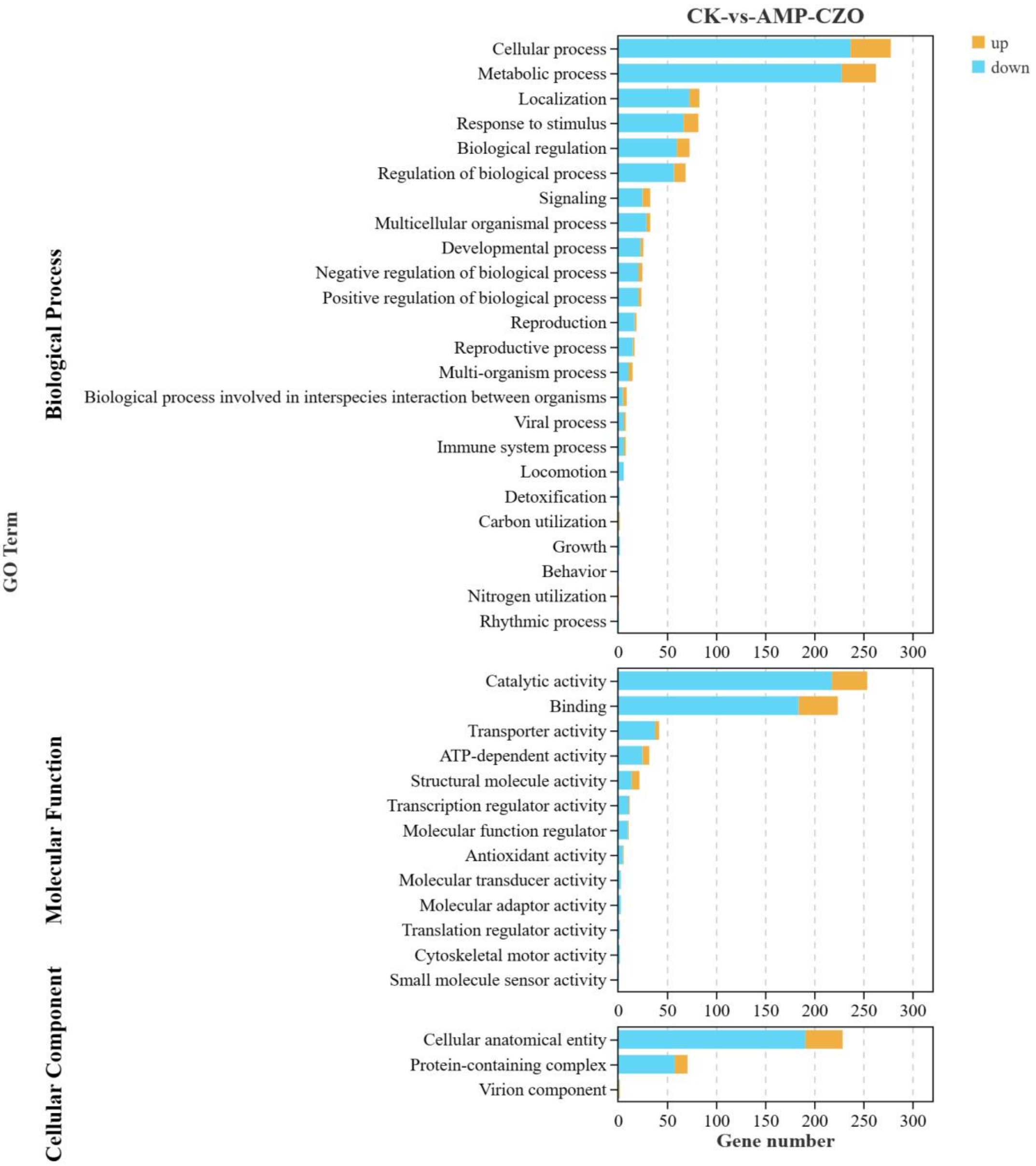

C

Figure S5 GO terms

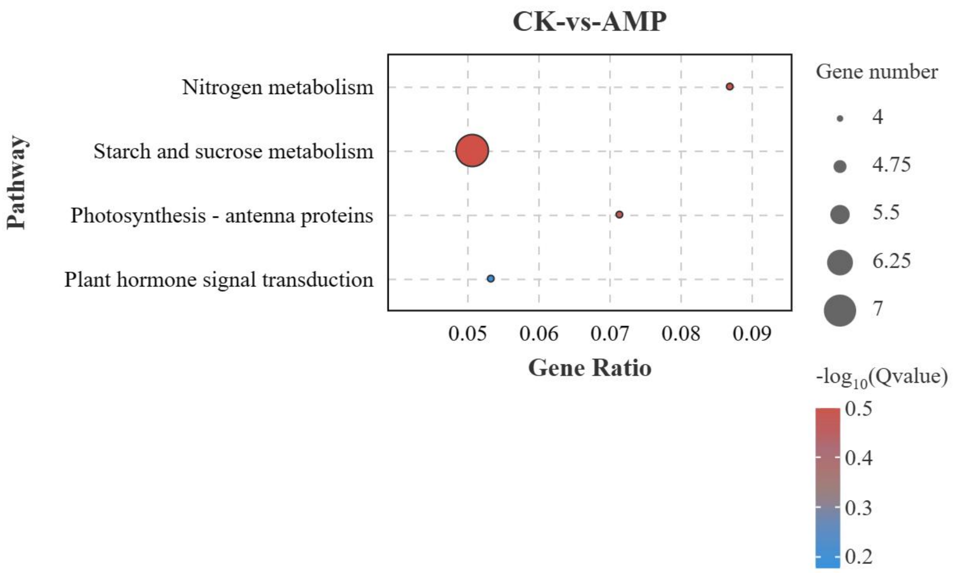
CK-vs-AMP
Pathway
Nitrogen metabolism
Starch and sucrose metabolism
Photosynthesis - antenna proteins
Plant hormone signal transduction
0.05
0.06
0.07
0.08
0.09
Gene Ratio
Gene number
4
4.75
5.5
6.25
7
-log10(Qvalue)
0.5
0.4
0.3
0.2

A

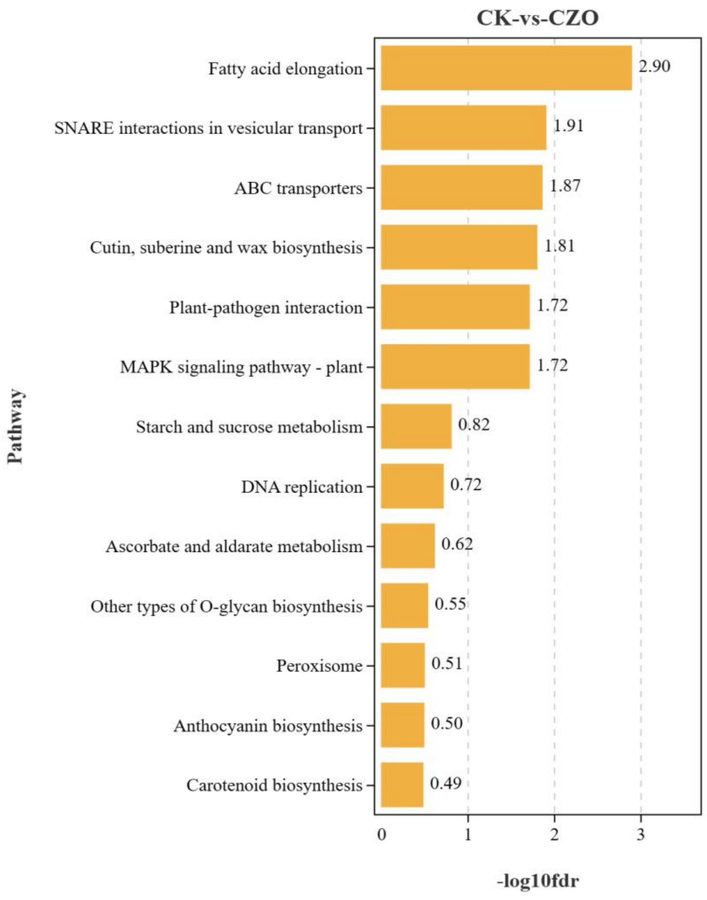
CK-vs-CZO
Pathway
Fatty acid elongation
2.90
SNARE interactions in vesicular transport
1.91
ABC transporters
1.87
Cutin, suberine and wax biosynthesis
1.81
Plant-pathogen interaction
1.72
MAPK signaling pathway - plant
1.72
Starch and sucrose metabolism
0.82
DNA replication
0.72
Ascorbate and aldarate metabolism
0.62
Other types of O-glycan biosynthesis
0.55
Peroxisome
0.51
Anthocyanin biosynthesis
0.50
Carotenoid biosynthesis
0.49
0
1
2
3
-log10fdr

B

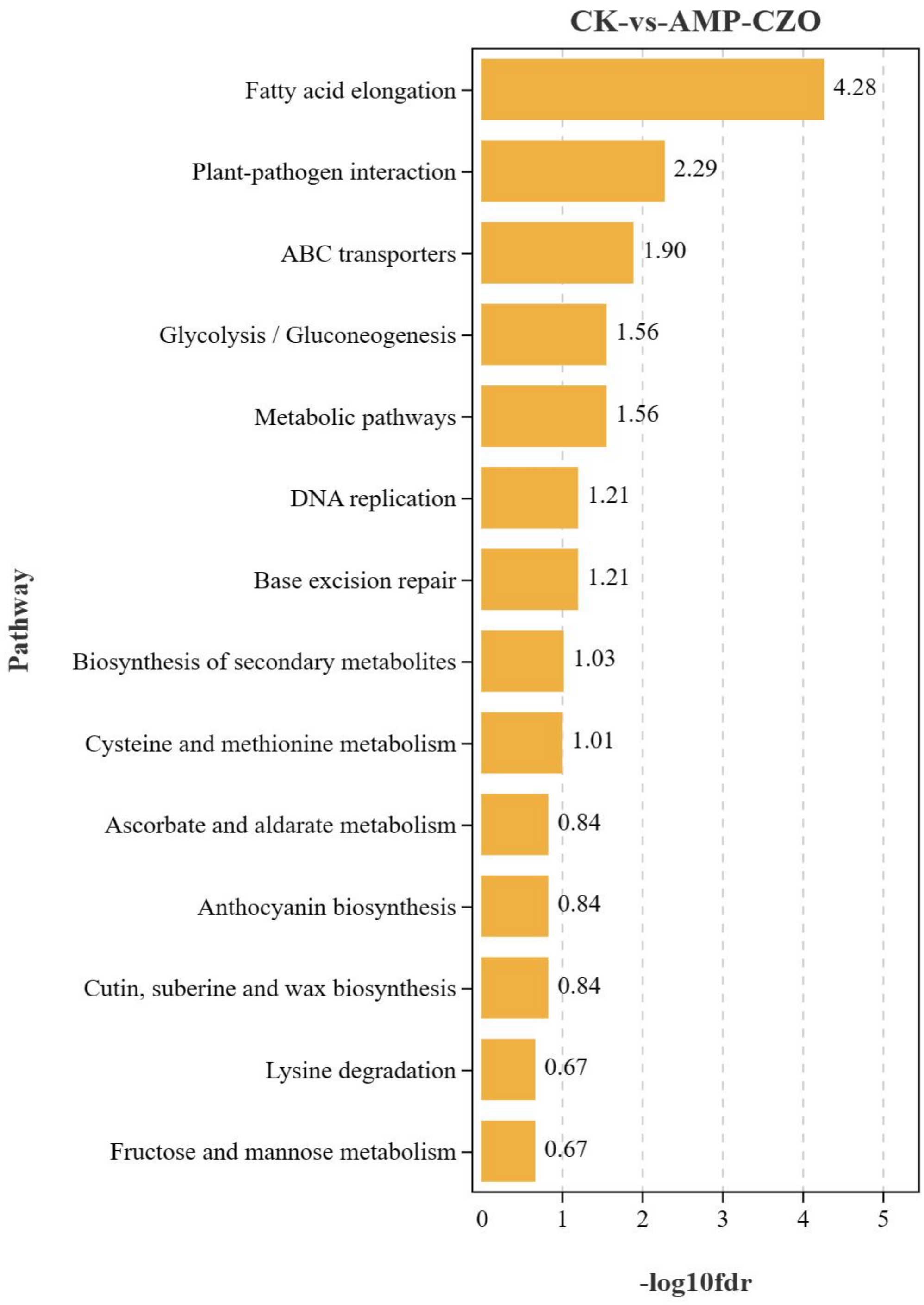

C

Figure S6 KEGG pathway
